# Ordered and deterministic cancer genome evolution after p53 loss

**DOI:** 10.1038/s41586-022-05082-5

**Published:** 2022-08-17

**Authors:** Timour Baslan, John P. Morris, Zhen Zhao, Jose Reyes, Yu-Jui Ho, Kaloyan M. Tsanov, Jonathan Bermeo, Sha Tian, Sean Zhang, Gokce Askan, Aslihan Yavas, Nicolas Lecomte, Amanda Erakky, Anna M. Varghese, Amy Zhang, Jude Kendall, Elena Ghiban, Lubomir Chorbadjiev, Jie Wu, Nevenka Dimitrova, Kalyani Chadalavada, Gouri J. Nanjangud, Chaitanya Bandlamudi, Yixiao Gong, Mark T. A. Donoghue, Nicholas D. Socci, Alex Krasnitz, Faiyaz Notta, Steve D. Leach, Christine A. Iacobuzio-Donahue, Scott W. Lowe

**Affiliations:** 1 Cancer Biology and Genetics Program, Memorial Sloan Kettering Cancer Center, New York, NY USA; 2 Computational and Systems Biology Program, Memorial Sloan Kettering Cancer Center, New York, NY USA; 3 Howard Hughes Medical Institute, Chevy Chase, MD USA; 4 Rubinstein Center for Pancreatic Cancer Research, Memorial Sloan Kettering Cancer Center, New York, NY USA; 5 PanCuRx Translational Research Initiative, Ontario Institute for Cancer Research, Toronto, Ontario Canada; 6 Cold Spring Harbor Laboratory, Cold Spring Harbor, NY USA; 7 Technical School of Electronic Systems, Technical University of Sofia, Sofia, Bulgaria; 8 Phillips Research North America, Oncology Informatics and Genomics, Cambridge, MA USA; 9 Molecular Cytogenetics Core Facility, Memorial Sloan Kettering Cancer Center, New York, NY USA; 10 Marie-Josee and Henry R. Kravis Center for Molecular Oncology, Memorial Sloan Kettering Cancer Center, New York, NY USA; 11 Dartmouth Cancer Center, Hanover, NH USA; 12 Present Address: Department of Pharmacology, University of North Carolina at Chapel Hill School of Medicine, Chapel Hill, NC USA; 13 Present Address: Lineberger Comprehensive Cancer Center, University of North Carolina at Chapel Hill, Chapel Hill, NC USA; 14 Present Address: Department of Pathology, Molecular and Cell-based Medicine, Icahn School of Medicine at Mount Sinai, New York, NY USA

**Keywords:** Cancer genetics, Cancer genomics

## Abstract

Although p53 inactivation promotes genomic instability^1^ and presents a route to malignancy for more than half of all human cancers^2,3^, the patterns through which heterogenous *TP53* (encoding human p53) mutant genomes emerge and influence tumorigenesis remain poorly understood. Here, in a mouse model of pancreatic ductal adenocarcinoma that reports sporadic p53 loss of heterozygosity before cancer onset, we find that malignant properties enabled by p53 inactivation are acquired through a predictable pattern of genome evolution. Single-cell sequencing and in situ genotyping of cells from the point of p53 inactivation through progression to frank cancer reveal that this deterministic behaviour involves four sequential phases—*Trp53* (encoding mouse p53) loss of heterozygosity, accumulation of deletions, genome doubling, and the emergence of gains and amplifications—each associated with specific histological stages across the premalignant and malignant spectrum. Despite rampant heterogeneity, the deletion events that follow p53 inactivation target functionally relevant pathways that can shape genomic evolution and remain fixed as homogenous events in diverse malignant populations. Thus, loss of p53—the ‘guardian of the genome’—is not merely a gateway to genetic chaos but, rather, can enable deterministic patterns of genome evolution that may point to new strategies for the treatment of *TP53-*mutant tumours.

## Abstract (web summary)

Malignant evolution enabled by p53 inactivation in mice proceeds through an ordered and predictable pattern of *Trp53* loss of heterozygosity, accumulation of deletions, genome doubling and the emergence of gains and amplifications.

## Main

Inactivating mutations in the *TP53* tumour suppressor gene are associated with cancers that are particularly aggressive and refractory to therapy^2,4^. Some of the earliest insights into p53 action and the consequences of *TP53* mutation linked p53 to a DNA-damage-induced cell cycle checkpoint of which the inactivation enables genomic instability^5–8^, implying that p53 acts as a guardian of the genome to prevent the emergence of cells containing potentially tumour-promoting mutations^9^. Subsequent research has demonstrated that p53 transcriptionally coordinates an expansive set of cell fate programs that actively limit tumorigenesis and the disruption of which remains critical for tumour maintenance^5,10–14^. However, owing to the absence of markers that discretely define cells after p53 inactivation, precisely how genomic instability manifests and shapes the transformation of *TP53*-mutant lineages as a consequence of the loss of these programs has not been defined.

Next-generation sequencing studies of human tumours associate *TP53* mutations with features of genomic instability, including rampant copy-number alterations (CNAs)^15^, chromothripsis^16^ and whole-genome doubling (polyploidy)^17,18^, and *TP53*-mutant tumours often display substantial intratumoral heterogeneity^19,20^. Consistent with a role for p53 inactivation as an enabler of genomic chaos, inferential reconstructions of the order of events from bulk sequencing data often places *TP53* mutations early in evolutionary time, preceding other genomic rearrangements^3^. However, the timing and order with which these features arise after *TP53* inactivation and their relationship with the biological transitions in stepwise cancer development have not been established, in part because human cancers are examined at the end point and not as they progress through the benign-to-malignant transition. Such information is important to understand the relationship between *TP53* mutations, genomic instability and cancer progression, and may ultimately inform therapeutic interventions.

*TP53* mutations are a prominent feature of pancreatic ductal adenocarcinoma (PDAC), a lethal disease also dominated by frequent mutations in other well-established driver genes including oncogenic mutations in *KRAS*  and inactivating mutations in the cell cycle inhibitor *CDKN2A* and/or the TGF-β pathway effector *SMAD4*^21^. The loss of *TP53* represents a key inflection point in the progression of PDAC, as *TP53* loss of heterozygosity (LOH) resulting in biallelic *TP53* inactivation is strongly associated with progression to invasive and genomically heterogeneous disease^22,23^ with recent studies linking acquired CNAs to disease progression and phenotypic heterogeneity^24,25^. Nevertheless, given the limited availability of patient tissue before and after tumour development^26,27^, it has been impossible to gain a temporal picture of how p53 inactivation leads to the evolution of PDAC genomes during malignant progression. Here we used a dual-fluorescence lineage-tracing model of PDAC that reports selection for *Trp53* LOH in vivo, thereby permitting the direct observation of the evolutionary dynamics of cells undergoing stepwise progression to malignancy at the single-cell resolution from the point of p53 inactivation. Our results demonstrate that tumour evolution after p53 inactivation in the setting of pancreatic transformation is not random but is subject to deterministic features that contribute to the genomic and biological hallmarks of *Trp53-*mutant tumours.

## Lineage tracing of sporadic *Trp53* LOH

**Extended Data Fig. 1:**
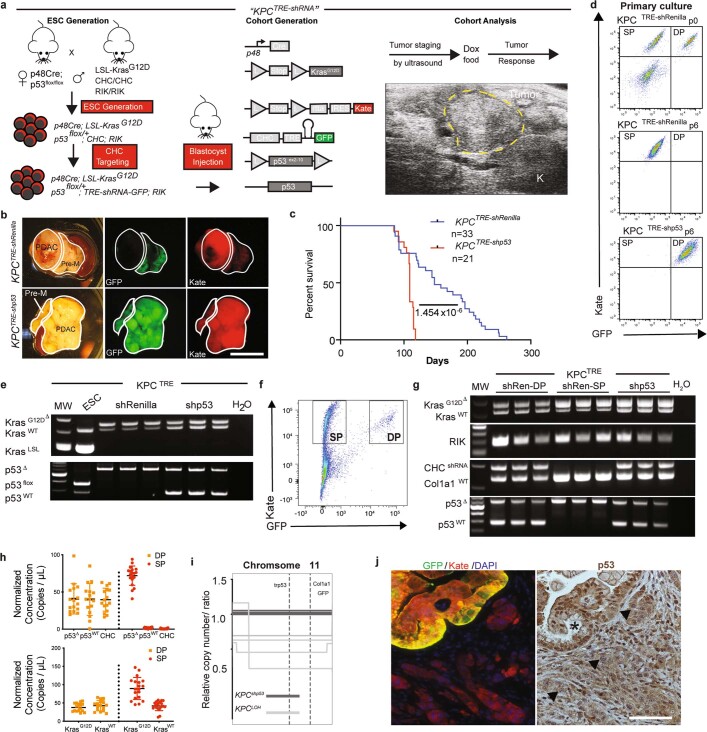
Fluorescent linkage reports sporadic p53 loss of heterozygosity in the KPC^LOH^ PDAC model. **a**, Schematic of breeding, embryonic stem cell engineering, allelic configuration, and staging of KPC^TRE-shRNA^ ESC PDAC. Dashed line defines tumour mass detected by ultrasound, K, kidney. **b**, Representative whole mount bright field and fluorescent gross pathology of PDAC arising in context of adjacent premalignant (Pre-M) tissue in KPC^shRenilla^ and KPC^shp53^ mice. **c**, Survival curve of KPC^TRE-shRenilla^ (n = 33) and KPC^TRE-shp53^ (n = 21) mice. Significance of difference in survival curves assessed by log rank (Mantel-Cox) test. **d**, Kate and GFP flow cytometry of primary cultures of dissociated shRenilla and shp53 KPC^TRE^ pancreas following PDAC development at indicated passages. **e**, PCR detection of recombined versus wild type alleles for Kras and p53 in primary cultures from indicated samples at passage 6. **f**, Representative flow cytometry plot distinguishing single Kate positive (SP) from double Kate/GFP positive (DP) cells after PDAC development in a KPC^shRenilla^ mouse. **g**, PCR detection of recombined versus wild type alleles for Kras, p53, as well as shRNA and RIK transgenes in DP and SP cells sorted from KPC^shRenilla^ mice following PDAC development and cell lines generated from PDAC arising in KPC^shp53^ mice. **h**, (Top) Digital PCR detecting relative levels of recombined conditional p53 allele, WT p53, and GFP targeted CHC cassette and (bottom) Kras^G12D^ and WT Kras alleles in DP (n = 16) and SP (n = 19) cells sorted from KPC^TRE-shRenilla^ PDAC. **i**, Relative copy number of chromosome 11 inferred from sparse whole genome sequencing from PDAC arising in KPC^shRenilla^ and KPC^shp53^ mice (n = 4 each). Normalized segment values are centred around a mean value of 1 with segment values below 1 indictive of deletion events. **j**, Representative, matched immunofluorescence of GFP and mKate and immunohistochemistry of p53 in sequential sections in a KPC^shRenilla^ PDAC. h, mean ± S.D. Scale bars b, 1 cm, j 50 μm. e and g were repeated at least twice with similar results, and j was repeated 3 times with similar results. Gel source data for e and g, see Supplementary Fig. 1. Gating strategy of d and f, see Supplementary Fig. 2. Source data

Mouse pancreas cancer models driven by conditional activation of an oncogenic *Kras*^*G12D*^ allele and a single conditional inactivating allele of *Trp53* (hereafter, the KPC model) result in pathophysiologically accurate PDAC that develops with near invariable loss of the remaining wild-type (WT) *Trp53* allele (hereafter *p53*)^28–30^. To expedite the production of experimental cohorts and enable stage-specific genetic perturbations, we developed a series of PDAC models that are produced directly from multiallelic embryonic stem cells (GEMM-ES cells)^31^. By integrating alleles that facilitate inducible expression of short hairpin RNAs (shRNAs) in pancreatic cells expressing mutant *Kras*, we studied the role of tumour-suppressor loss in PDAC maintenance and chromatin regulation in early-stage neoplasia^10,32,33^. To extend this approach to PDAC initiated by mutant *Kras* and mono-allelic inactivation of p53, we generated a GEMM-ES cell platform containing the following alleles: a pancreas-specific *cre*, a lox-stop-lox *Kras*^*G12D*^ allele, a conditional knockout *p53* allele (*p53*^*flox*^), a lox-stop-lox *rtTA-IRES-mKate* allele knocked into the *Rosa26* locus^34^ and a collagen homing cassette (*CHC*)^35^ introduced into the *Col1a1* gene that facilitates targeting of various genetic elements using recombination-mediated cassette exchange (Extended Data Fig. 1a and Methods).

**Fig. 1:**
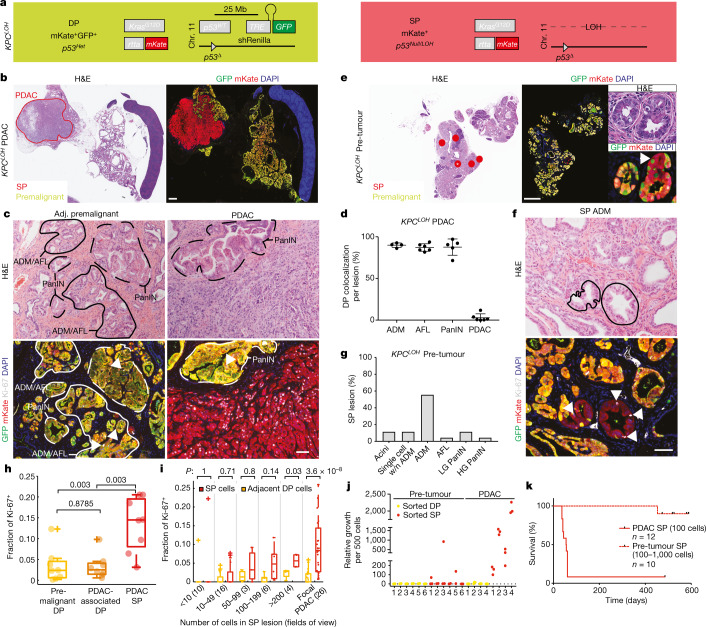
Lineage tracing of incipient cancer cells after sporadic p53 inactivation in mouse PDAC. **a**, Schematic of *KPC*^*LOH*^: fluorescent tracking of *p53* LOH in *Kras*-driven pancreatic tumorigenesis. **b**, Representative haematoxylin and eosin (H&E) staining (left) and mKate/GFP immunofluorescence (IF, right) of SP (mKate^+^) versus DP (mKate^+^GFP^+^) cells in a PDAC-bearing (red outline) mouse. **c**, Representative H&E staining (top) and Kate/GFP/Ki-67 immunofluorescence (bottom) in the adjacent (Adj.) premalignant tissue (left) versus focal PDAC (right). The solid outline indicates ADM and AFL. The dashed outline shows PanIN. The arrowheads indicate Ki-67^+^ cells. **d**, DP cell frequency in ADM, AFL, PanIN and PDAC. *n* = 6. **e**, Representative H&E (left) and mKate/GFP immunofluorescence (right) of SP (red dots) versus DP cells in a mouse without PDAC. Inset: H&E (top) and immunofluorescence (bottom) analysis of SP cells within a DP structure (indicate by an asterisk (*)). **f**, Representative H&E (top) and Kate/GFP/Ki-67 immunofluorescence (bottom) analysis of ADM SP lesions (solid lines) observed in a mouse without PDAC. The arrowheads indicate Ki-67^+^ SP cells. **g**, Characterization of SP lesions in *KPC*^*LOH*^ mice without PDAC. *n* = 43 lesions, *n* = 7 mice. HG, high grade; LG, low grade; w/n, within. **h**, The percentage of Ki-67^+^ DP and SP cells in adjacent premalignant and PDAC tissue. *n* = 8. **i**, The percentage of Ki-67^+^ SP and DP cells in lesions of the indicated size in *KPC*^*LOH*^ mice without frank PDAC. *n* = 9. **j**, The relative growth of 500 DP or SP cells sorted before (pre-tumour, *n* = 6) and after (PDAC, *n* = 4) frank PDAC development. **k**, The survival of mice transplanted with 100–1,000 SP cells sorted from *KPC*^*LOH*^ mice with (solid line, 12 injections, 6 each from 2 mice) or without (dashed line, *n* = 10) frank PDAC. For **b** and **e**, the experiments were repeated at least three times with similar results. For **d**, data are mean ± s.d. For the box plots in **h** and **i**, the centre line shows the median, the box limits show the 25th and 75th percentiles, and the whiskers show the range; outliers are shown. For **h** and **i**, significance was assessed using two-tailed Wilcoxon's rank-sum tests. Scale bars, 1 mm (**b** and **e**) and 50 μm (**c** and **f**). Source data

**Extended Data Fig. 2:**
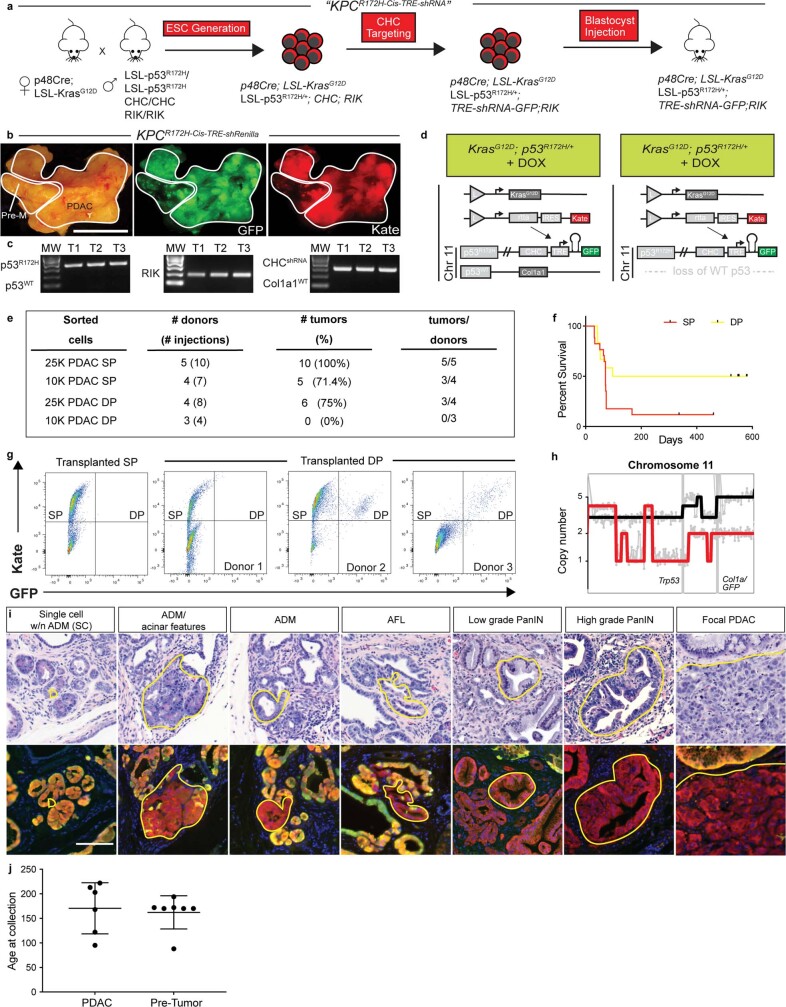
Linkage in cis with mutant p53 retains inducible shRNA expression following p53 LOH in the KPC^Cis-shRNA^ PDAC model and functional and histological characterization of the premalignant to malignant transition captured by stage dependent analysis of *KPC*^*LOH*^ mice. **a**, Schematic of breeding, embryonic stem cell engineering, allelic configuration, and cohort generation of the KPC^R172H-CIS-TRE-shRNA^ ESC PDAC GEMM. Note that founder LSL-p53^R172H^; CHC double homozygotes were utilized to ensure segregation of the conditional mutant p53 allele in cis with the collagen homing cassette. **b**, Representative whole mount bright field and fluorescent gross pathology of PDAC and adjacent premalignant (Pre-M) tissue in KPC^R172H-Cis-TRE-shRenilla^ mice. **c**, PCR detecting recombination of the conditional p53^R172H^ allele versus WT p53 allele (left), RIK allele (centre), and targeted CHC versus WT Col1a1 allele (right) in primary cancer cell lines derived from PDAC developing in KPC^R172H-Cis-TRE-shRenilla^ mice (n = 3). Note the absence of WT p53 and WT Col1a1, but the maintenance of the targeted CHC allele. **d**, Schematic depiction of maintenance of GFP linked shRNA in cis with the conditional mutant p53^R172H^ allele during p53 LOH and PDAC progression in KPC^R172H-Cis-TRE-shRNA^ mice. **e**, Tabular results of tumours developing after injection of indicated number of DP and SP cells sorted from PDAC bearing KPC^LOH^ mice into immunocompromised, nude mice. **f**, Survival curve of injected recipients detailed in e. **g**, Flow cytometry of GFP and Kate in a representative tumour resulting from injection of PDAC associated SP cells (left) and flow cytometry of tumours resulting from injection of 25000 DP cells sorted from 3 of 4 mice as indicated in e. Tumours were composed of either exclusively SP cells (Donor 1), predominantly SP cells (Donor 2), or exclusively DP cells (Donor 3). **h**, Absolute copy number of chromosome 11 inferred from sparse whole genome sequencing from GFP positive tumours (as shown in C) arising from cells with focal p53 deletion. Red and black segments denote diploid and polyploid tumours respectively. **i**, Histological characterization of SP lesions (yellow outlines) observed before frank PDAC development in “Pre-tumour” *KPC*^*LOH*^ mice. These mice are defined by the lack of clear tumour development by ultrasound. **j**, Age at collection of Pre-tumour (n = 7) and PDAC (n = 6) *KPC*^*LOH*^ mice subjected to single cell genomic analysis. j, mean ± S.D. Scale bars. b, 1cm, i, 50 μm. c was repeated at least twice with similar results. Gel source data for c, see Supplementary Fig. 1. Gating strategy for g, see Supplementary Fig. 2. Source data

In developing this platform, we produced a model that did not function as initially intended but instead offered the ability to trace the lineage of cells that have lost p53 function (Fig. 1a and Extended Data Fig. 1). Owing to the linkage of the *CHC* to the WT *p53* allele on the opposite chromosome as the conditional *p53* allele, the GFP-coupled shRNA cassette is lost as PDAC develops after *p53* LOH (Fig. 1a). Thus, in a setting in which a neutral GFP-linked shRNA (here, for example, targeting *Renilla* luciferase (shRenilla)) is incorporated into the system and the mice are fed doxycycline chow, premalignant tissue is double-positive (DP) for mKate (that is, lineage tracing of cells upon mutant Kras activation) and GFP (shRNA) fluorescence, whereas the resulting PDAC, which acquires *p53* LOH, is single positive (SP) for mKate fluorescence (Fig. 1a and Extended Data Fig. 1b). Incorporation of a GFP-coupled *p53* shRNA into the *CHC* locus to suppress *p53* in *trans* prevented the loss of GFP fluorescence in PDAC, demonstrating that the transition from DP to SP cells results from selection for p53 inactivation (Extended Data Fig. 1b–e). Genotyping and digital PCR of sorted DP versus SP cells, along with sparse whole-genome sequencing (WGS) and immunohistochemistry (IHC) analysis of pancreatic tissue after PDAC development confirmed the genomic and functional link between the loss of the GFP-positive *CHC* locus and WT *p53* (Extended Data Fig. 1f–j). GFP retention in PDAC was also observed using a complementary model in which a hot-spot *p53* mutant (*p53*^*R172H*^) was engineered in *cis* to the *CHC* and not lost after *p53* LOH (*KPC*^*cis-shRNA*^; Extended Data Fig. 2a–d). Thus, the physical linkage between the *p53* locus and a fluorescent reporter in this model (designated *KPC*^*LOH*^) acts as a lineage-tracing mark of *p53* LOH, permitting phenotypic analysis of cells after selection for biallelic *p53* inactivation.

Analysis of *KPC*^*LOH*^ pancreata after PDAC development confirmed that SP cells (acquiring *p53* LOH) have malignant properties, whereas DP cells (retaining WT *p53*) do not. Thus, SP tumour cells displayed a malignant histology, whereas DP cells were confined to lesions with premalignant histopathology irrespective of their location within the tumour mass or adjacent premalignant tissue (Fig. 1b–d). In agreement, SP cells sorted from PDAC tissue had a much greater tumour-initiating potential compared with tumour-associated DP cells after orthotopic transplantation into immunocompromised mice (Extended Data Fig. 2e,f), and the few tumours that arose from DP cells had no GFP fluorescence (for example, representing cells that underwent a *p53* LOH event) or focal *p53* alteration events that maintained the GFP targeted locus (Extended Data Fig. 2g,h).

We envisioned that the lineage-tracing abilities of the above model would enable in vivo genotyping of cells throughout PDAC progression. Notably, an analysis of tissues derived from *KPC*^*LOH*^ mice lacking detectable PDAC (that is, pre-tumour) revealed that SP cells were present as cells emerging within DP structures, or as variably sized lesions histologically consistent with premalignant cell fate: acinar to ductal metaplasia (ADM), atypical flat lesions (AFL), and low- and high-grade pancreatic intraepithelial neoplasia (PanIN)^36–38^ (Fig. 1e–g and Extended Data Fig. 2i). In contrast to the high proliferative fraction and tumour-initiating potential of SP cells present in frank PDAC, SP cells within premalignant lesions showed a low proliferative fraction that increased with lesion size and, when isolated from mice without gross PDAC, displayed poor colony-forming and tumour-initiating abilities (Fig. 1h–k). Thus, these results imply that selection for *p53* loss in pancreatic cells expressing oncogenic *Kras* is not sufficient in and of itself to confer malignant fitness, but that these properties are acquired over time, facilitated by the absence of p53 function. As such, the *KPC*^*LOH*^ model enables the isolation of p53-deficient cells at distinct stages of transformation (Extended Data Fig. 2i,j), including an initial, intermediate evolutionary phase that connects p53 inactivation to the acquisition of cancer-initiating potential.

## CNAs follow *p53* LOH

**Fig. 2:**
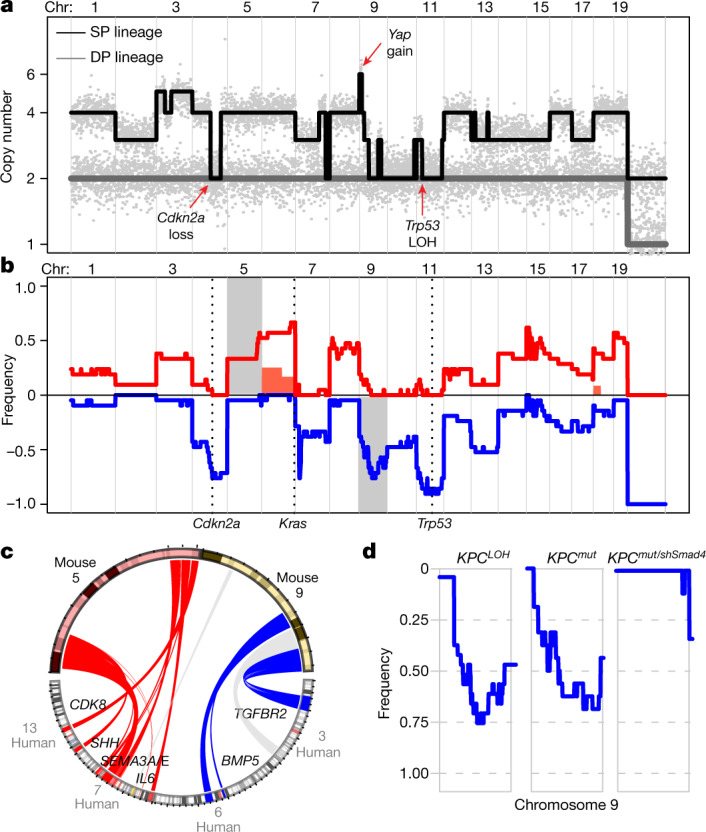
Recurrent and conserved CNAs targeting PDAC drivers shape the evolution of malignant genomes after p53 inactivation. **a**, Matching genome-wide copy-number profiles of SP and DP cells isolated from a polyploid *KPC*^*LOH*^ PDAC. The red arrows indicate distinguishing alterations. **b**, Frequency plot of recurrent CNAs from sequencing-sorted DP (*n* = 14) and SP (*n* = 24) cells after PDAC development. The chromosomes highlighted in grey denote regions recurrently altered in SP samples and analysed for synteny with human PDAC data. The filled red trace denotes chromosome 6 gains found in a subset of DP samples. The vertical dashed lines denote the location of PDAC driver genes. **c**, Human–mouse synteny Circos rendering of selected alterations on mouse chromosomes 5 and 9. The red and blue colouring denotes gains and deletions with matching species synteny, respectively. The grey colouring denotes no matching genomic intervals in directionality (for example, gains or loss in both species). Selected PDAC-relevant genes are shown. **d**, Chromosome 9 deletion frequency plot in *KPC*^*LOH*^ (*n* = 22), *KPC*^*mut*^ (*n* = 16) and *KPC*^*mut/shSmad4*^ mouse PDACs (*n* = 7). Chr, chromosome.

**Extended Data Fig. 3:**
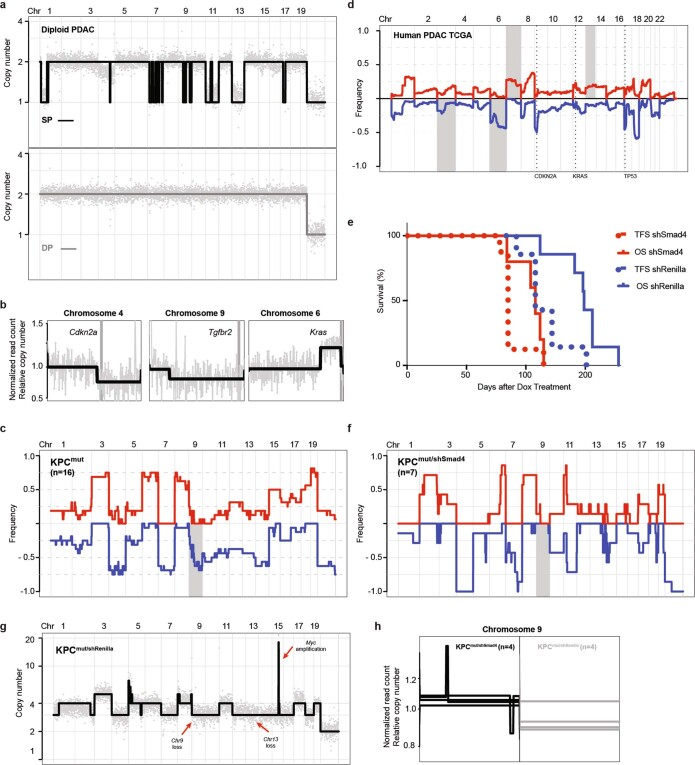
Genome evolution following p53 inactivation is characterized by the emergence of recurrent and conserved copy number alterations that target known PDAC drivers. **a**, Representative genome-wide copy number profile of SP and DP cellular populations isolated from a diploid KPC^LOH^ PDAC. **b**, Representative Zoom-in chromosomal views of copy number alterations acquired in KPC^shp53^ PDAC mice. Normalized segment values are centred around a mean value of 1 with segment values below and above 1 indictive of deletion and gain events, respectively. Vertical grey bar denotes location of PDAC driver gene. **c**, Frequency plot of acquired copy number events in 16 cell lines derived from PDACs arising in mice harbouring the hotspot p53 allele; R172H. Grey bar denotes chromosome 9 which encodes regulators of the TGF- β pathway. **d**, Frequency plot of copy number landscape of human PDAC (TCGA). Dotted black lines denote location of driver genes; TP53, KRAS, CDKN2A. Grey bar denotes location of recurrent events that are syntenic to chromosomes found altered in KPC^LOH^ PDAC genomes. **e**, short hairpin mediated suppression of Smad4 in KPC^cis/shRNA^ accelerates disease onset and is associated with worse survival. OS = overall survival, TFS = tumour free survival. Log rank p values for TFS is 0.0051 and OS is 0.0016. **f**, Frequency plot of copy number landscape of cancer cell lines from KPC^cis-shSmad4^ mice. Grey bar denotes chromosome 9 loss which is alleviated via shSmad4 perturbation. **g**, Genome-wide copy number profile of a tumour arising in KPC^mut/shRenilla^. Arrows denote distinguishing genomic alterations including deletion of chromosome 9. **h**, Zoom-in-view of chromosome 9 relative copy number in tumours arising in KPC^mut/shSmad4^ and KPC^mut/shRenilla^ (n = 4 each).

The genomes of *TP53-*mutant cancers are already highly rearranged and genomically heterogenous at diagnosis^15,19^. To determine the specific consequence of p53 inactivation on genomic evolution during PDAC development, we performed sparse WGS comparing 38 flow-sorted SP and DP populations isolated from PDAC-bearing pancreata, including 17 matched DP and SP pairs. Whereas DP genomes were invariably diploid and rarely displayed CNAs, SP cell genomes were highly rearranged and frequently polyploid (Fig. 2a and Extended Data Fig. 3a). Notably, consistent with biological selection for additional genomic driver events, SP cells sorted after PDAC development acquired recurrent losses on chromosomes 4, 7, 9, 11 and 13, as well as gains on chromosomes 3, 5, 6, 8 and 15 (Fig. 2b). These alterations were also observed in sequencing profiles obtained from PDAC produced by shRNA-mediated p53 suppression or after *p53* LOH in cells containing a recurrent *p53* hotspot mutation (such as *KPC*^*R172H*^), implying that copy-number evolution in *p53* altered PDAC results from p53 inactivation and does not require gain-of-function effects of mutant *p53* (Extended Data Fig. 3b,c).

A meta-analysis of recurrent gains and losses in sorted SP populations from PDAC-bearing mice (*n* = 24) revealed conservation with CNAs that are frequently observed in human PDAC. These conserved CNAs possess known PDAC drivers, such as deletions on chromosomes 4 and 11 (encompassing the *Cdkn2a* and *p53* loci, respectively) as well as gains of chromosome 6, including *Kras*^39^ (Fig. 2b and Extended Data Fig. 3d). Other recurrent CNAs that occurred in both species include deletions of chromosome 9 (corresponding to regions on human chromosomes 3 and 6) and gains of chromosome 5 (corresponding to human chromosomes 7 and 13) (Fig. 2b,c and Extended Data Fig. 3d). These regions contained genes implicated in processes that are linked to PDAC development, including chromatin remodelling (*Mll2* and *Setd2*), axon guidance (*Sema3a* and *Sema3b*), PDAC proliferation or progression (*Il6*, *Shh* and *Cdk8*) and TGF-β signalling (*TgfbrII* and *Bmp5*)^40–43^.

The presence of recurrent copy-number events encompassing known PDAC drivers and their synteny to those present in *TP53*-mutant human tumours implies that the selective forces driving genome evolution in *TP53-*mutant cancers are similar across species. To test whether these acquired events target functionally relevant pathways, we enforced one predicted consequence of chromosome 9 deletions—that is, TGF-β pathway disruption—using shRNA-mediated knockdown of *Smad4*, the transcriptional effector of TGF-β signalling, in the *KPC*^*cis-shRNA*^ model described above (Methods and Extended Data Fig. 2). *Smad4* suppression not only accelerated the development of PDAC with inactivated p53, but also alleviated the pressure to lose chromosome 9, effects that were not observed in otherwise identical cohorts with a neutral (shRenilla) shRNA (Fig. 2d and Extended Data Fig. 3e–h). Notably, the effects of *Smad4* suppression may alter selection for copy-number changes beyond chromosome 9 loss, as the deletion landscape in *KPC*^*cis-shSmad4*^ tumours also exhibits altered frequencies of losses on chromosomes 4, 12 and 13 (Extended Data Fig. 3f–h). Thus, recurrent deletions can target critical pathways that contribute to the phenotypic and genomic evolution of *p53*-mutant cancers.

## Ordered phases of genome evolution

The above data validate the *KPC*^*LOH*^ model as a powerful platform to link the acquisition of genomic rearrangements to the phenotypic progression to malignancy after p53 inactivation. As our genomic and functional analyses nominate recurrent CNAs as such selected events, we performed single-cell genome sequencing of lineages defined by p53 inactivation isolated from *KPC*^*LOH*^ mice both after cancer development and during the benign-to-malignant transition (namely, PDAC versus pre-tumour mice; Extended Data Fig. 2j). We reasoned that such an approach would enable us to visualize the accumulation of CNA events over time and leverage the nature of the acquired CNAs and their associated breakpoints as an additional lineage-tracing dimension to establish detailed phylogenetic relationships during distinct phases of tumour evolution after p53 loss (Methods).

**Fig. 3:**
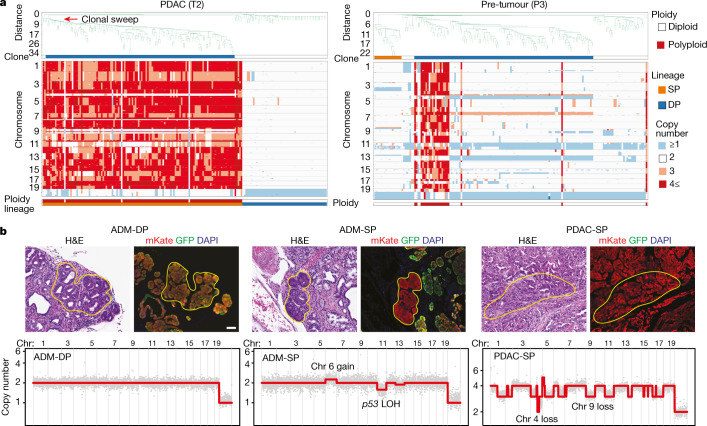
Distinct and ordered phases of genome evolution accompany the benign-to-malignant switch. **a**, Breakpoint-based phylogenetic tree of single SP (*n* = 130) and DP (*n* = 55) cells sequenced from PDAC sample T2 (left). The red arrow indicates a split in the neighbour-joining tree and clonal sweep of SP cells. Distance is based on statistical considerations of breakpoint similarity/dissimilarity (Methods). Sweeping SP cells share a clonal relationship with a false-discovery rate (FDR) not exceeding a threshold value of *t* = 0.01. Right, breakpoint-based phylogenetic tree of single SP cells (*n* = 171) sequenced from pre-tumour sample P3. The clone track denotes a lineage that underwent genome doubling (navy). The clonal relationship between diploid and polyploid cells is computed with an FDR not exceeding a threshold value of *t* = 0.01. Colour codes for ploidy, lineage and copy number are provided. **b**, Matched H&E and immunofluorescence of lesions that underwent LMD (yellow outlines) (top). Bottom, matched copy-number profiles of lesions collected by LMD. Scale bar, 50 μm.

**Extended Data Fig. 4:**
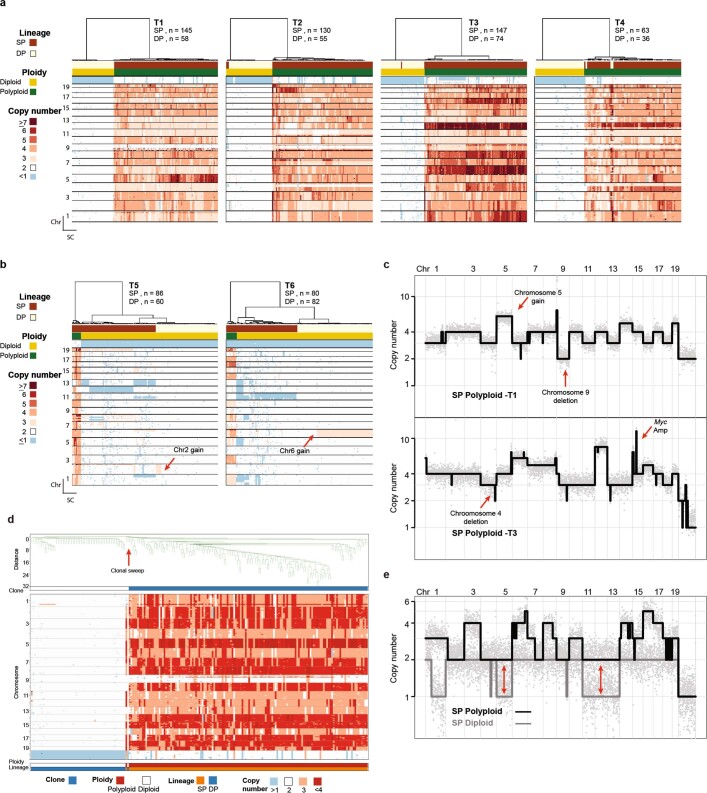
Single-cell genome analysis after PDAC development reveals two discrete genomic states distinguishing DP and SP populations. **a**, Hierarchal, copy number clustering heatmaps of SP and DP single cells sequenced from 4 polyploid PDAC. Colour code for lineage (L), ploidy (P), and chromosome copy number are provided. Sample annotation and number of single-cells sequenced are provided. **b**, Hierarchal, copy number clustering heatmaps of SP and DP single cells sequenced from 2 diploid PDAC. Colour code for lineage (L), ploidy (P), and chromosome copy number are provided. Sample annotation and number of single-cells sequenced are provided. Red arrows point to alterations acquired in DP cells that are not observed in matching SP cells. **c**, Genome-wide copy number profiles of representative SP single cells sequenced from PDAC samples T1 and T3 illustrating p53 null rearranged genomes. Red arrows indicate selected recurrent alterations. **d**, Breakpoint based phylogenetic tree of single SP (n = 130) and DP (n = 55) cells sequenced from PDAC T1. Colour codes for ploidy, lineage, and copy number information are provided. Red arrow points to split in the neighbour-joining tree demarcating the clonal sweep of SP cells. Phylogenetic distance is based on statistical considerations of breakpoint similarity/dissimilarity (Methods). SP cells constituting the sweeping clone share a clonal relationship with a False Discovery Rate (FDR) not exceeding a threshold value of *t* = 0.01. **e**, Matched genome wide copy number profiles of diploid and polyploid SP cells sequenced from T5. Red arrows indicate shared common alterations designating clonal relationships between cells.

Single-cell sequencing of DP and SP cells from six PDAC-bearing pancreata (designated T1–T6) corroborated the bulk sequencing data of flow-sorted populations and permitted the analysis of intratumoural genetic heterogeneity and the clonal relationships between *p53*-intact and *p53-*LOH lineages (Fig. 3a and Extended Data Fig. 4a–e). As expected, DP cells were largely euploid without recurrent CNAs. Although in two cases a subset of DP cells had gains on chromosomes 2 and 6, matched SP cell populations lacked these gains (Extended Data Fig. 4b). By contrast, SP cells from PDAC-bearing mice (hereafter, PDAC-SP cells) carried a large number of CNAs, were genomically heterogeneous and mostly polyploid (Fig. 3a and Extended Data Fig. 4a,c). Breakpoint-based phylogenetic analysis (Methods) revealed that this intratumoural heterogeneity was often associated with a clonal sweep of related polyploid PDAC-SP cells (for example, PDAC samples T1 and T4) that lacked a definable relationship with matched diploid DP cells (Fig. 3a and Extended Data Fig. 4d). Thus, single-cell sequencing of PDAC tissue from the *KPC*^*LOH*^ model reveals two discrete genomic states defined by *p53* status without an apparent evolutionary medium to connect them (namely, DP, diploid and non-rearranged; SP, polyploid and highly rearranged).

**Extended Data Fig. 5:**
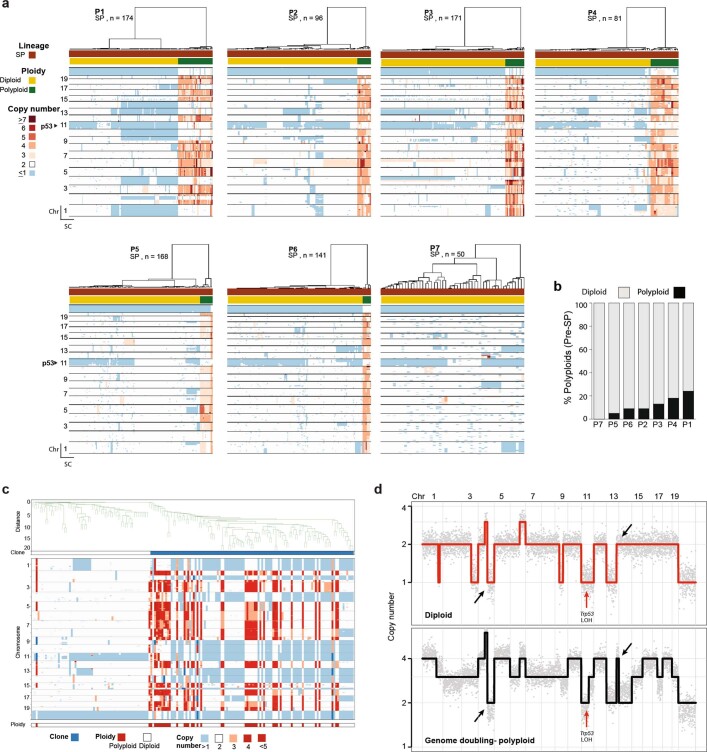
Single-cell genome sequencing of SP cells from Pre-tumour mice reveals an intermediate evolutionary genomic state. **a**, Hierarchal, copy number clustering heatmaps of SP single cells sequenced from 7 Pre-tumour mice. Colour code for lineage (L), ploidy (P), and chromosome copy number are provided. Sample annotation and number of single-cells sequenced are provided. **b**, Bar-plot quantification of percent SP cells sequenced that were polyploid across Pre-tumour mice analysed. **c**, Breakpoint based phylogenetic tree of single SP cells (n = 171) sequenced from Pre-mouse P1. Colour codes for ploidy, lineage, and copy number information are provided. Phylogenetic distance is based on statistical considerations of breakpoint similarity/dissimilarity (Methods). Clone track denotes lineage that underwent genome doubling (purple). Clonal relationship between diploid and polyploid single cells is computed with a FDR not exceeding a threshold value of *t* = 0.01. **d**, Genome-wide copy number profiles of representative single cells of highly rearranged diploid Pre-SP cells (top panel) and its genetically traced polyploid counterpart (top panel) from Pre-Tumour Sample P3. Black arrows denote distinguishing copy number alterations and their breakpoint positions used in inferring phylogenetic relationships.

**Extended Data Fig. 6:**
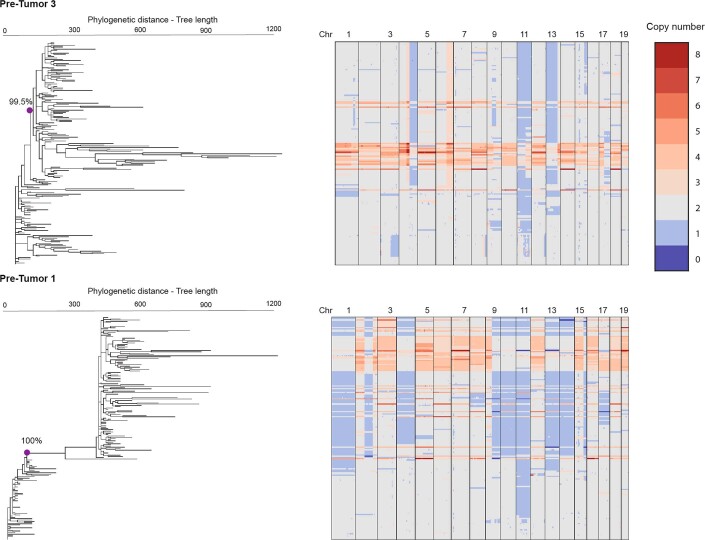
Phylogenetic tree inference of genome doubling timing based on copy number, minimal event distance metric. Phylogenetic reconstruction of diploid and polyploid single cells sequenced from Pre-Tumour 3 (top) and Pre-Tumour 1 (bottom) based on minimum event distance (MED) metric as described by Kauffman et al. Phylogenetic tree and associated heatmaps are depicted. Each branch corresponds to a single sequenced cell. Purple circle indicates node in the tree where diploid and polyploid cells share a branching relationship. Statistic adjacent to circle denotes branching support values calculated via 200 bootstrap resampling iterations. Algorithm provided support tree panels with bootstrap confidence statistics on branch/node relationships are provided in Supplementary Fig. 4 and 5 for Pre-Tumour 1 and 3, respectively.

Analysis of SP cells from pre-tumour mice provided a bridge. Single-cell analysis of SP cells isolated from seven age-matched non-tumour-bearing mice (hereafter, pre-tumour SP cells, P1–P7) revealed that pre-tumour SP populations were distinct from PDAC SP and associated DP cells in that they remained largely diploid but had acquired a wide-range of CNAs (Fig. 3a and Extended Data Fig. 5a). Moreover, a small subpopulation of polyploid cells was detected in 6 out of the 7 pre-tumour SP samples analysed and, in most cases, could be related to a diploid precursor (Fig. 3a and Extended Data Figs. 5b–d and 6). Thus, single-cell sequencing establishes distinct phases of genome evolution after *p53* LOH in which CNAs are first acquired in diploid cells, with polyploidy emerging as a relatively late event. Consistent with this evolutionary continuum, two PDAC-SP populations presented as a mixture of rearranged diploid and related polyploid cells (Extended Data Fig. 4b,e).

**Extended Data Fig. 7:**
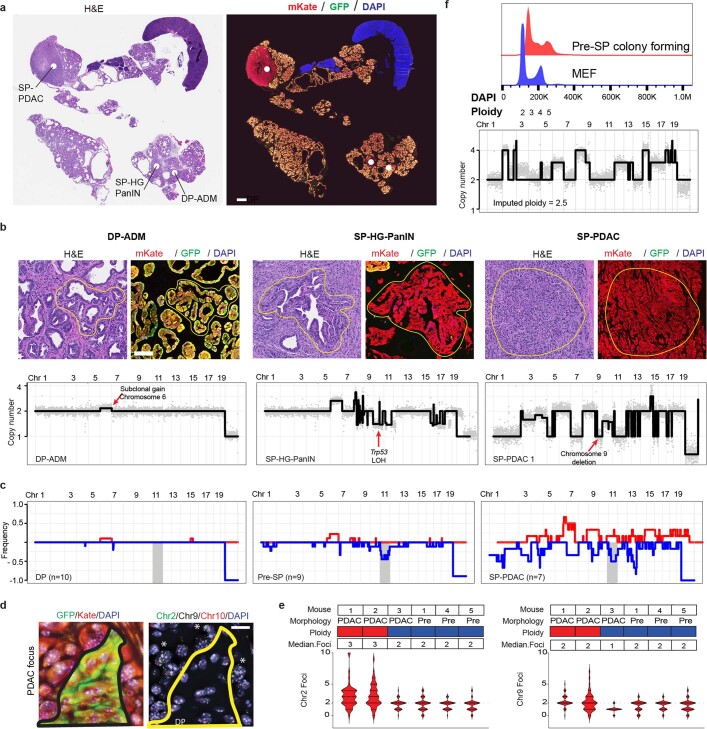
*In-situ* genomic analysis directly links level of genome rearrangement with histopathological phenotypes during PDAC progression. **a**, Matched H&E and immunofluorescence for mKate/GFP in sequential sections after PDAC development in a KPC^LOH^ mouse. White circles denote positions of SP and DP lesions with premalignant morphology and SP cells with PDAC morphology subjected to laser microdissection (LMD). **b**, Top panels - images of microdissected lesions noted in (a) - yellow lines denote boundaries of LMD. H&E as well as IF images are displayed. Bottom panels - corresponding genome wide copy number profiles of microdissected premalignant and malignant lesions. Red arrows denote distinguishing copy number alterations. **c**, Frequency plot of aggregate lesions collected by LMD and sequenced for each category. DP, n = 10; Pre SP, n = 9; SP-PDAC, n = 7. **d**, Matched GFP and Kate immunofluorescence and DNA FISH of chromosome 2, 9, and 10 in DP and SP cells within a focus of PDAC. Asterisks indicate cells with FISH signals consistent with polyploidy and loss of chromosome 9. **e**, Quantification of DNA-FISH foci in SP cells identified in KPC^LOH^ mice before frank PDAC development (n = 5). For details of quantification, see Methods. **f**, DAPI based flow cytometric nuclear profiling of sorted Pre-SP cells capable of colony formation when plated at low density in-vitro (from Fig. 1j) (top-panel) and corresponding copy number profile with the sequencing imputed ploidy for the sample (bottom panel). MEF; Mouse Embryonic Fibroblasts. Scale bars a 1 mm, b 50 μm, d 10 μm. Gating strategy for f, see Supplementary Fig. 2. Source data

The above results were confirmed by in situ genomic analysis of pathologically defined benign and malignant lesions. Specifically, we performed fluorescence guided laser microdissection (LMD) followed by sequencing of DP and SP lesions isolated from tumour and pre-tumour mice (Methods). Consistent with bulk and single-cell sequencing data, DP cells were largely euploid containing occasional gains on chromosome 6, whereas PDAC-SP lesions invariably acquired widespread copy-number changes and were frequently polyploid (Fig. 3b and Extended Data Fig. 7a–c). Pre-SP lesions with premalignant morphology were diploid and contained few CNAs (mainly deletions), with the loss of chromosome 11 (where *p53* resides) being the dominant event (Fig. 3b and Extended Data Fig. 7b,c). DNA FISH analysis confirmed that prominent chromosome 9 deletions and polyploidy were restricted to SP cells with PDAC histopathology (Extended Data Fig. 7d,e). Consistent with these histological findings, the majority of rare pre-tumour SP cells capable of colony formation in vitro displayed rearranged polyploid genomes (Extended Data Fig. 7f). These results illustrate phases of genomic evolution that directly couple the degree of CNA acquisition and ploidy state after *p53* LOH to malignant pancreatic transformation.

## Determinism governs evolutionary paths

**Fig. 4:**
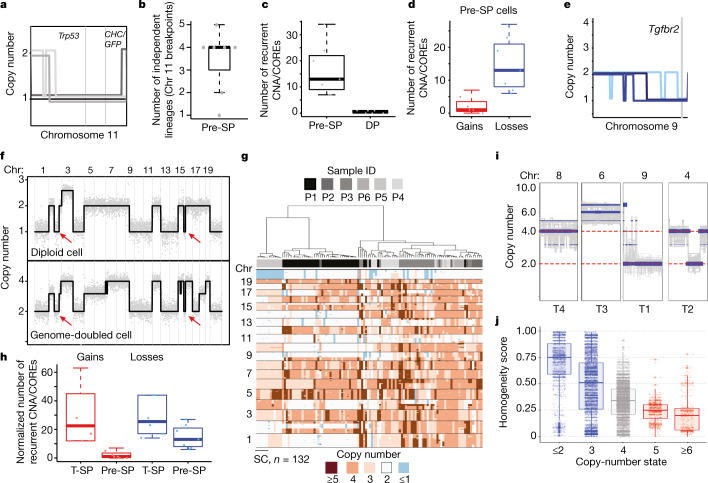
Deterministic principles govern the selection of genomic rearrangements after *p53* LOH. **a**, Breakpoints in LOH cells from sample P2 associated with chromosome 11 deletion reflecting the lineage heterogeneity of cells undergoing LOH events. **b**, Quantification of distinct *p53* LOH/chromosome 11 deletion breakpoints in 7 *KPC*^*LOH*^ pre-tumour mice. **c**, Quantification of acquired CNAs in SP cells from pre-tumour mice (*n* = 7) compared with DP premalignant cells (*n* = 6). Statistical analysis was performed using a two-tailed Mann–Whitney *U*-test; *P* = 0.00338. **d**, Quantification of CNAs identified in pre-SP cells from seven mice according to CNA class. Statistical analysis was performed using a two-tailed Mann–Whitney *U*-test; *P* = 0.0041. **e**, Recurrent chromosome 9 deletions identified in pre-SP cells. Distinct deletion events are uniquely coloured. The vertical grey line marks the location of *Tgfbr2*. **f**, Genome-wide copy-number profiles of a polyploid single cell and its inferred diploid precursor illustrating the genomic relationship and genome doubling. The diagonal red lines denote CNA-associated breakpoints used to infer lineage (Extended Data Fig. 5). **g**, Heat-map analysis of all of the identified polyploid pre-SP cells (*n* = 132) in pre-tumour mice (*n* = 7). P1 and P5 illustrate instances in which the emerging polyploid lineage is diversifying genomically. **h**, Quantification of CNA events per class (that is, deletion versus gain) in SP cells sequenced from tumour (*n* = 6) and pre-tumour mice (*n* = 7). Statistical analysis was performed using a two-sided *t*-test for enrichment of gains in polyploid cells; *P* = 0.005. **i**, Illustration of the heterogeneity/homogeneity of selected recurrent gains and deletions in *KPC*^*LOH*^ PDACs. The segments (blue lines) at multiple- or single-copy-number states indicate heterogeneity and homogeneity, respectively. **j**, Quantification of CNA segment homogeneity (Methods) based on single-cell copy-number data of SP cells from PDAC mice. *n* = 4. For **c**, **d**, and **h**, recurrent CNAs were computed using the algorithm CORE (Methods). Box plots are as defined in Fig. 1. Source data

**Extended Data Fig. 8:**
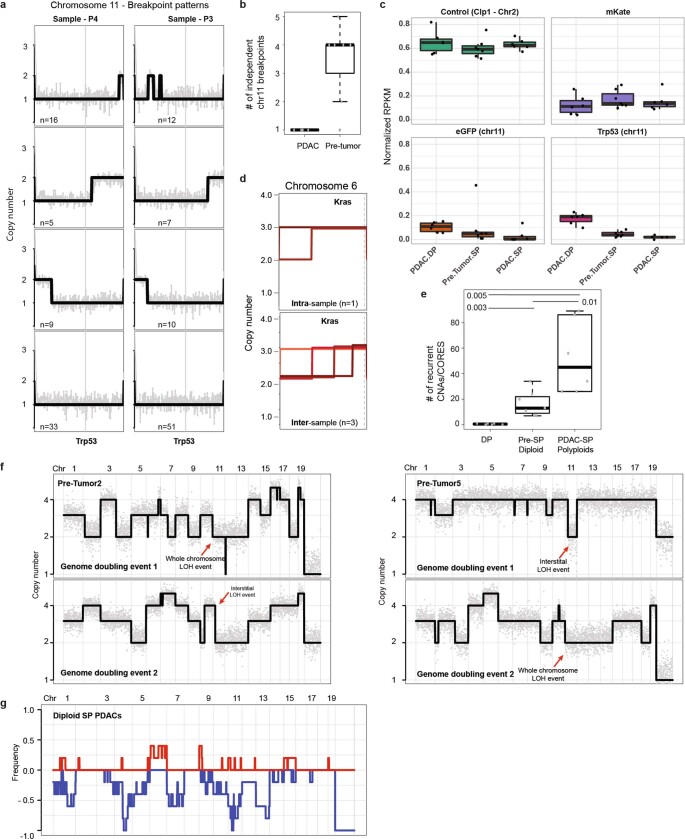
Selective and ordered patterns of genome evolution during cancer initiation. **a**, Unique breakpoint patterns associated with chromosome 11 deletions identify independent p53 LOH lineages. Grey dots illustrate normalized raw read count values. Black lines illustrate segmented data. Vertical grey line denotes location of Trp53. Number of single cells sequenced representing each unique breakpoint event is provided. **b**, Boxplot quantification of unique lineages based on chromosome 11 deletion breakpoints in Pre-SP diploid cells (n = 7 samples) compared to PDAC-SP polyploid cells (n = 4 samples). Mann-Whitney U two-sided test of significance for number of LOH events in Pre-tumour and PDAC-SP samples, p-value = 0.03. **c**, Boxplot quantification of normalized read count mappability data from a census single cell genotyping approach (Methods) from PDAC-DP (n = 6), PDAC-SP (n = 6), and Pre-tumour SP (n = 7) single-cell sequencing at eGFP, Trp53, mKate, and Clp2 (control) sequences. **d**, Zoom in chromosomal views illustrating intra- (from within one animal) and inter- (between different animals) alteration heterogeneity of Kras gains (chromosome 6). **e**, Boxplot quantification of acquired copy number alterations detected in single-cell sequencing data from DP (n = 6), Pre-SP diploids (n = 7), and PDAC-SP samples (n = 6). p-value < 0.05 for all pairwise two-sided Mann-Whitney U test of significance with exact values provided in figure. **f**, Genome-wide illustration of independent genome doubling events observed in polyploid cells from non-tumour bearing sample P1 (left panels) and P2 (right panels). Red Arrows denote distinguishing p53 LOH events. **g**, Frequency plot depiction of diploid SP PDACs from the KPC^LOH^ model (n = 7). Box plots are as defined in Fig. 1. Source data

Further examination of the single-cell data revealed non-random patterns through which copy-number changes are selected during discrete phases of genome evolution after p53 inactivation. Pre-tumour SP cells displayed distinct breakpoint patterns on chromosome 11, indicative of independent, competing, *p53* LOH lineages emerging during the benign-to-malignant switch (Fig. 4a,b). A single-cell census genotyping approach confirmed that these resulted in loss of the WT *p53* haplotype (Fig. 4a,b and Extended Data Fig. 8a–c). Although some pre-tumour SP cells had only chromosome 11 deletions, evolving populations gradually acquired additional deletions, including recurrent events conserved in mouse and human PDAC. The one exception to this deletion-centric pattern involved occasional interstitial gains of chromosome 6 encompassing *Kras* that, owing to their distinct structural features compared with those occurring in DP cells, were most likely acquired after *p53* LOH (Figs. 2a–c and 4c–e and Extended Data Fig. 8d,e). These results add granularity to a previous report implying that *Kras* gains contribute to tumorigenesis after p53 inactivation^39^. Thus, the most proximal events to p53 inactivation involve the accumulation of deletions in diploid cells, including functionally validated deletions on chromosome 9 (for example, TGF-β signalling; Fig. 2).

Of the 10–20% of pre-tumour SP cells that were polyploid, a breakpoint-based phylogenetic analysis demonstrated that most could be traced to highly rearranged diploid precursors (Figs. 3a and 4f,g and Extended Data Figs. 5c,d and 6). In some instances, different genome doubling events arising from distinct diploid precursors were detected while, in others, a single event was followed by rapid genomic diversification resulting in a heterogenous expanding polyploid lineage (Fig. 4g and Extended Data Fig. 8f). These results are consistent with genome doubling as an active process (for example, occurring multiple times during the evolution of *p53* LOH lineages) and, when giving rise to expanding polyploid clones, arising from highly rearranged diploid precursors (Figs. 3a and 4f,g and Extended Data Fig. 5).

**Extended Data Fig. 9:**
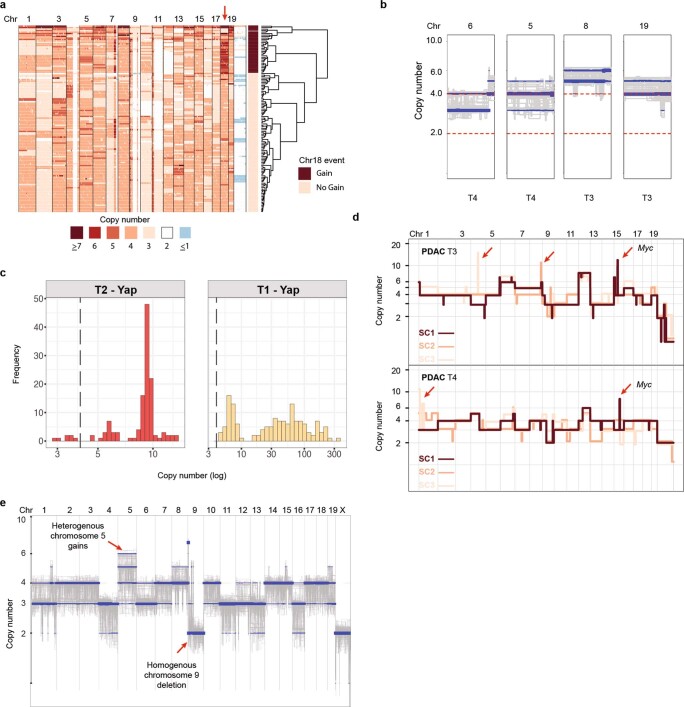
Genomic heterogeneity of acquired gains and amplifications in KPC^LOH^ PDAC. **a**, Hierarchal clustering tree of copy number profiles from tumour 2 (T2). Subpopulation of cells enriched for subclonal chromosome 18 gain are annotated on bar underneath the clustering dendrogram. Red vertical arrow indicates alteration. **b**, Zoom in chromosomal view of sub-clonal gains found in PDAC sequenced from KPC^LOH^ model at single-cell resolution. Dashed red lines communicate reference copy number states for diploid and polyploid genomes, two and four, respectively. **c**, Histogram illustration of copy number values of YAP amplifications identified in two PDAC samples sequenced at single-cell resolution. Histograms illustrate heterogeneity of YAP amplifications. **d**, Overlay of three representative single-cell, genome-wide copy number profiles derived from two sequenced PDACs. Diagonal red arrow indicates amplifications identified in only a single sequenced cell. **e**, Genome-wide aggregate plot of all single-cells sequenced from KPC^LOH^ PDAC T1. Thickness of blue line is proportional to percentage of cells carrying a given alteration at a copy number state. Red arrows point to alterations on chromosome 5 (gain) and 9 (deletion) found heterogeneously and homogenously, respectively.

Although polyploid pre-tumour SP cells continued to acquire deletion events, they also began to accumulate widespread chromosomal gains and focal amplifications that were largely absent in the diploid state (Fig. 4g,h). In agreement, polyploid PDAC-SP cells also displayed more gains and amplifications compared with diploid pre-tumour SP and PDAC-SP cells (Fig. 4h and Extended Data Fig. 8g). Although many of these gains and amplifications encompassed validated oncogenic drivers, they were invariably subclonal and displayed three layers of heterogeneity: (1) presence or absence in a subclone; (2) variation in copy-number state between related cells; and (3) single focal events that target validated drivers such as *MYC*^44^ (Fig. 4i and Extended Data Fig. 9a–d). By contrast, deletion events maintained a higher degree of homogeneity compared with gains both at recurrent deletion events (for example, chromosome 9) as well as at the genome-wide level (Fig. 4i,j and Extended Data Fig. 9e). These results establish a deterministic pattern of genome evolution during pancreatic neoplasia: *p53* LOH, followed by the accumulation of deletions, polyploidy and then gains and amplifications. They also imply that polyploidy enables the accumulation of a broader repertoire of CNAs that, in the case of chromosomal gains, are generally not tolerated in cells with diploid genomes.

## Conservation of patterns in human PDAC

**Fig. 5:**
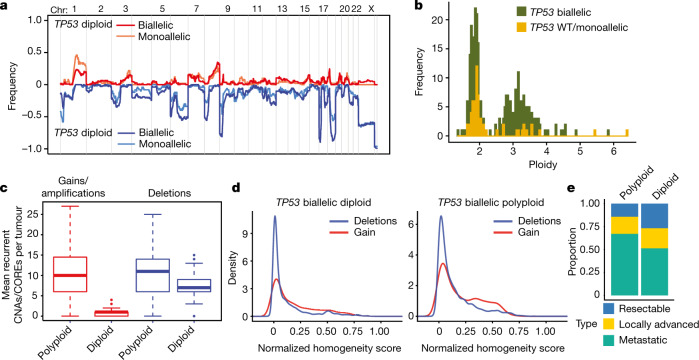
Whole genomes, targeted capture and single-cell sequencing corroborate evolutionary principles in human disease. **a**, The copy-number landscape of diploid *TP53* biallelic PDAC compared with diploid *TP53*-mono/WT PDAC from the COMPASS dataset. **b**, PDAC ploidy according to *TP53* allelic state from COMPASS dataset. *TP53* biallelic mutant PDAC are significantly more likely to exhibit polyploidy. Statistical analysis was performed using the Fisher exact test; *P* = 10^−6^. **c**, Quantification of CNA events, as computed using the algorithm CORE (Methods) per class (that is, deletion versus gain) in all polyploid (*n* = 137) and diploid (*n* = 156) human PDACs from the COMPASS trial. Statistical analysis was performed using a two-sided *t*-test for gain/amplification enrichment in polyploid cells; *P* = 2.2 × 10^−16^. **d**, Kernel-density estimation of normalized homogeneity (Methods) of CNAs genome wide from targeted capture (MSK-IMPACT) of PDAC (*n* = 1,076 total) cases according to ploidy and *TP53* mutation status. Chromosomal gains/amplifications are significantly more likely to be heterogenous. Statistical analysis was performed using a two-sample Kolmogorov–Smirnov test; *P* < 0.005. Empirical cumulative distribution function measurements are shown in Extended Data Fig. 10. **e**, Disease type in polyploid and diploid PDAC with biallelic *TP53* inactivation from the MSK-IMPACT dataset. Statistical analysis was performed using a Fisher exact test; *P* = 0.003. Box plots are as defined in Fig. 1. Source data

**Extended Data Fig. 10:**
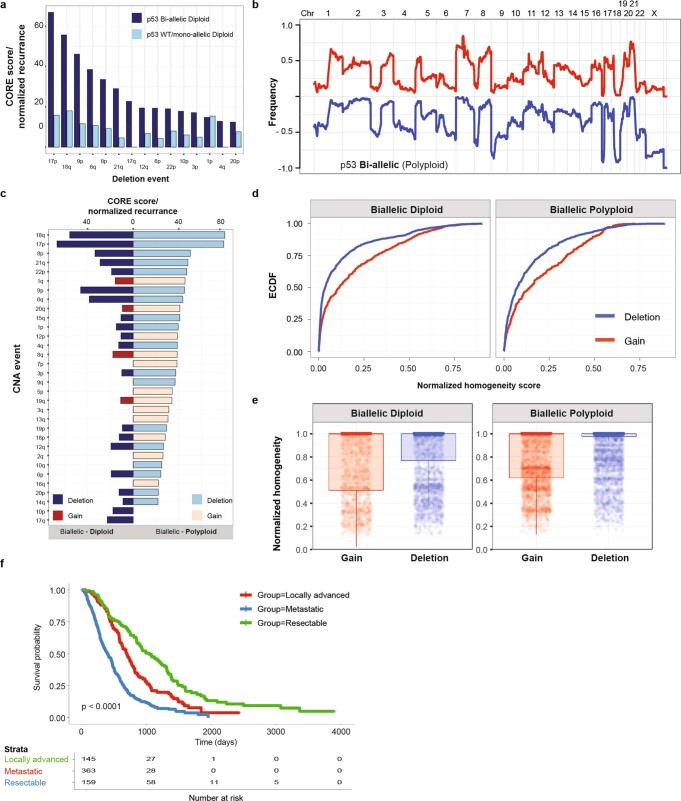
Whole genomes and targeted capture sequencing corroborates evolutionary principles in human PDAC. **a**, Bar-plot rendering of recurrent deletion event frequency as computed using the algorithm CORE (Methods) comparing p53 bi-allelically mutant vs. p53 mono-allelic and wild type (WT) diploid PDAC in COMPASS dataset. Cytoband of recurrent events is depicted on X-axis. Events were selected based on a threshold p-value of < 0.005. **b**, Frequency plot illustration of acquired copy number alterations in polyploid, p53 bi-allelically mutant human PDAC in COMPASS dataset. **c**, Bar-plot quantification of the frequency of recurrent CNAs (blue for deletion and red for gain) in polyploid PDAC genomes compared to diploid genomes in COMPASS dataset. Alterations are rank ordered according to CORE score. A p-value of < 0.005 was used as cut-off threshold for event inclusion. **d**, Empirical Cumulative Distribution Function (ECDF) for CNA event in p53 bi-allelically mutant diploid (left) and polyploid (right) datasets from MSK-IMPACT PDAC datasets. **e**, Bar-plot depiction of normalized homogeneity score for deletion and gain event type in the COMPASS dataset based on cell fraction and purity estimates from FACETs algorithm. **f**, Cox-regression survival of clinically annotated MSKCC PDACs samples. Metastatic cases, which are enriched for p53-biallelic polyploid samples have worst survival, p-val < 0.001.

**Extended Data Fig. 11:**
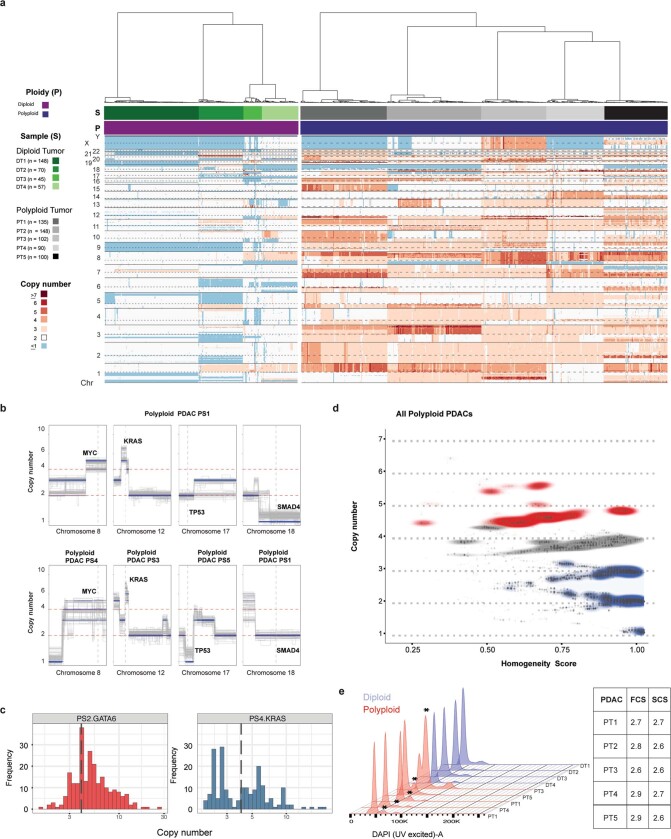
Single cell sequencing corroborates evolutionary principles in human PDAC. **a**, Copy number based hierarchal clustering heatmap of 9 human PDAC (4 diploid and 5 polyploid) sequenced at single-cell genome resolution. Ploidy, Sample ID, and copy number colour schema are provided. **b**, Chromosomal zoom-in-view of aggregate single-cell segments from polyploid tumours illustrating heterogeneity of amplifications at MYC and KRAS, contrasting with the homogeneity of deletion events at TP53 and SMAD4. **c**, Histogram of copy number values across sequenced single cells for selected amplicons found in two different polyploid PDAC cases. Vertical dashed line denotes reference copy number state of 4. **d**, Normalized homogeneity score (Single cell homogeneity score – Methods) of copy number alterations across all polyploid single cells sequenced according to copy number state. Red colouring denotes gains and amplifications. Blue colouring denotes deletions. Grey colouring denotes references state (copy number = 4). **e**, Flow cytometric measurements of nuclear DNA content (e.g. ploidy) in human PDAC samples analysed at single-cell resolution. Asterisk denotes the polyploid population from which single-cells were gated and sorted. Median ploidy values of gated polyploid populations compared to sequencing inferred ploidy values are tabulated. FCS; flow cytomeric. SCS; single-cell sequencing.

Analysis of human PDAC using whole-genome sequencing^25^, targeted capture sequencing (MSKCC-IMPACT)^45^ and single-cell sequencing datasets confirmed that the genomes of *TP53*-mutant PDAC display patterns predicted from our lineage-tracing model (Fig. 5 and Extended Data Figs. 10 and 11). Consistent with an initial deletion-centric route to genome evolution, diploid PDAC sustaining biallelic *TP53* mutations contained more recurrent deletions (for example, 9p, 17p, 18q) compared with those retaining one or two copies of WT *TP53* and showed a relative paucity of gains and amplifications (Fig. 5a and Extended Data Fig. 10a). Furthermore, while tumours retaining WT *TP53* were invariably diploid, the majority of those harbouring biallelic *TP53* mutations were polyploid and had acquired substantially more gains and amplifications (Fig. 5b,c and Extended Data Fig. 10b,c).

**Extended Data Fig. 12:**
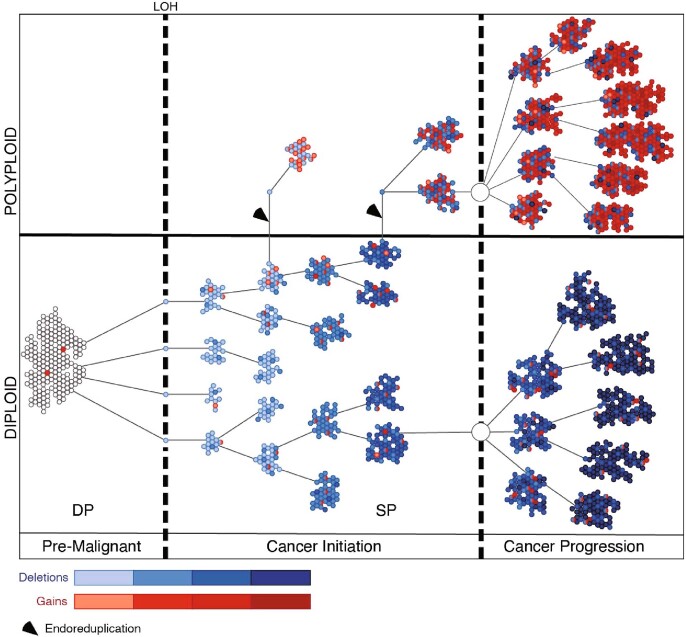
Schematic illustration of the deterministic principles governing the evolution of p53 mutant pancreatic cancer genomes. DP; double positive, p53^wt/flox^, SP; single positive, p53^wt/−^. Vertical dashed line illustrates point at which independent LOH clones emerge (first line) and selective sweeps that result in cancer progression (second line). Black arrow heads point to independent genome doubling events that can occur during transformation. Colour codes for deletion and gain CNAs are provided. Colour gradation is proportional to number of events acquired.

Analysis of bulk tumour samples indicated that deletion events exhibited a higher degree of homogeneity compared with gains in both diploid and polyploid genomes (Fig. 5d and Extended Data Fig. 10d,e), a result confirmed by single-cell sequencing of a series of diploid and polyploid tumours (Extended Data Fig. 11a,b). Thus, regions containing known tumour suppressors on chromosome 9p, 17p and 18q were found homogenously, whereas amplifications targeting *MYC*, *KRAS* and *GATA6*—oncogenic events that drive metastatic progression and/or influence PDAC subtypes^25,39,44^—were heterogenous (Extended Data Fig. 11c–e). Accordingly, patients with polyploid versus diploid PDAC with biallelic *TP53* mutations were significantly more likely to present with metastatic disease (Fig. 5e), which was associated with the worst survival in patients (Extended Data Fig. 10f). These results reinforce the deterministic evolutionary patterns observed in our mouse model, with early and homogenous deletion events dominating the diploid state and the emergence of polyploidy, gains and amplifications linked to more aggressive disease (Extended Data Fig. 12).

## Discussion

Despite the well-established association between p53 inactivation and genomic instability^1^, the trajectories by which genomic instability arises and shapes tumour progression after *TP53* loss have remained obscure owing to challenges in simultaneously monitoring *TP53* status, genome evolution and cellular phenotype during the stepwise process of malignant progression. Here we took advantage of a mouse pancreatic cancer model containing a unique reporter system to trace genome evolution after sporadic p53 inactivation during a previously inaccessible phase of cancer evolution initiated by p53 loss of function—at the benign-to-malignant transition. By pinpointing and tracing cells after *p53* LOH in premalignancy, we demonstrate that the evolution of malignant pancreatic genomes enabled by *p53* disruption is not random; instead, it occurs through distinct, ordered phases that operate with predictive principles that can be linked to specific histological stages and contribute functionally to tumour progression. Thus, although p53 inactivation unleashes rampant intratumoral heterogeneity, selective forces lead to a surprisingly reproducible pattern of genome evolution that can be observed in the corresponding human disease.

The lineage-tracing approach provides granularity that reveals a notable degree of determinism in the sequence of events, with deletions dominating early evolution and gains and amplifications being acquired later on. The preponderance of deletions as the earliest events after p53 inactivation in mouse and human PDAC implies that reduced activity of certain pathways (for example, TGF-β) may be essential for malignant initiation, whereas the increased activity of other pathways (such as MYC, axon guidance) may be more important during progression^46^. This ordered evolution is consistent with the ability of acute p53 inactivation to facilitate the acquisition of chromosome losses^47^ and suggests that deletions inferred as occurring early in sequencing and phylogenetic studies of cancer evolution^3,48,49^ may represent events involved in establishing fitness that drives premalignant-to-malignant transitions.

Our results also provide unanticipated insights into the emergence of polyploidy during tumorigenesis unleashed by p53 inactivation. Although associations between polyploidy and genome evolution with *TP53* mutation have been noted previously^18,48,49^, lineage tracing of pancreas cancer evolution from premalignancy through frank cancer development suggests that genome doubling neither precedes p53 inactivation nor is the first selected event after *p53* loss. Instead, *p53*-deficient cells accrue an excess of genomic deletions before genome doubling, after which newly polyploid cells diversify, enabling selection for chromosomal gains and amplifications. Thus, the initial selection for polyploidy may reflect an adaptive mechanism to compensate for rampant loss of gene dosage created by an excess of deletions, only then accelerating genome diversification, and the accumulation of oncogenic events linked to elevated gene dosage that apparently fuel tumour progression^25^. Indeed, we note that patients with *TP53*-mutant polyploid PDAC show a greater incidence of metastasis at diagnosis, a property that has recently been associated with *KRAS* and *MYC* amplifications in functional studies and in patients^25,39,44^.

Overwhelming evidence indicates that p53 suppresses tumorigenesis by inducing a set of transcriptionally regulated effector programs that collectively limit the proliferation of oncogene-expressing cells and of which the ongoing inactivation is needed to sustain disease^5,10–14^. However, by capturing p53-deficient cells well before the emergence of frank PDAC, we observed that *p53* loss is not sufficient in and of itself for malignancy; instead, the acquisition of recurrent CNAs is also required. Interestingly, although *p53*-deficient cells gain proliferative potential during the benign-to-malignant switch, the fact that this was not immediately detected may reflect the limitations of our assay or that other niche-specific factors (such as immune surveillance) create an initial selective advantage for *p53*-deficient cells. Regardless, our results provide compelling evidence that both the loss of canonical tumour suppressor functions and the ensuing genomic instability each functionally contribute to the emergence of aggressive cancer in our PDAC model.

Patients with *TP53*-mutant tumours have a poor prognosis and often respond poorly to cancer therapy^4^. The fact that *TP53* mutations are associated with polyploid tumours littered with CNAs and other rearrangements helps to explain the rampant heterogeneity that is a hallmark of *TP53* mutant cancers, undoubtedly contributing to their aggressive tumour behaviour^15,18,19^. At the same time, the predictable pattern of genome evolution enabled by p53 inactivation may have therapeutic ramifications. For example, although much focus has been placed on targeting oncogenes in amplified/gained regions, their subclonal nature in *TP53*-mutant polyploid tumours suggests that such strategies will eventually fail owing to a reservoir of resistant non-altered cells within the tumour mass. Instead, therapeutic approaches that exploit tumour-cell vulnerabilities created by homogeneous deletion events^50^ that arise before genome doubling may be a more effective (although challenging) approach to create durable responses in this patient population.

## Methods

### Development of KPC PDAC GEMM-ES cell models

*p48cre;LSL-Kras*^*G12D*^*;p53*^*flox/WT*^*;CHC;Rosa26-CAGGS-LSL-rtta-IRES-mKate2 (RIK)* embryonic stem (ES) cells were derived from embryonic day 3.5 (E3.5) blastocysts collected from superovulated, 3–6-week-old female *p48cre;p53*^*flox/flox*^ mice bred with 6–10 week-old male *LSL-Kras*^*G12D/+*^*;CHC/CHC;RIK/RIK* mice as previously described^31^. This breeding scheme results in segregation of the *CHC* and *p53*^*flox*^ loci in *trans* configuration on separate alleles of mouse chromosome 11. *p48cre;LSL-Kras*^*G12D*^*;p53*^*LSL-R172H/WT*^*;CHC;RIK* ES cells were derived similarly from E3.5 blastocysts collected from superovulated, 3–6-week-old female *p48cre;LSL-Kras*^*G12D*^ mice bred with 6–10-week-old male *p53*^*LSL-R172H/LSLR172H*^*;CHC/CHC;RIK/RIK* mice. This breeding scheme results in linkage of the *LSL-p53*^*R172H*^ and *CHC* loci in *cis* on mouse chromosome 11. In brief, blastocysts were incubated in an 80 μl drop of KSOM + AA (Millipore) under mineral oil for 7–8 h, washed briefly in M2 medium (Millipore) and cultured and passaged in KOSR + 2i medium using ESGRO Complete Accutase (Millipore) on irradiated DR4 mouse embryonic fibroblast feeder layers until expanded for cryopreservation and genotyping. Male *p48cre;LSL-Kras*^*G12D*^*;p53*^*flox/WT*^*;CHC;RIK* and *p48cre;LSL-Kras*^*G12D*^*;p53*^*LSL-R172H/WT*^*;CHC;RIK* ES cells were further expanded for targeting in M15 + LIF medium.

The *KPC*^*shRenilla/LOH*^ GEMM-ES cell mouse model was developed through FLP-mediated recombination of an shRNA targeting *Renilla* luciferase (guide strand: TAGATAAGCATTATAATTCCT, cloned into the cTGM vector^32^ into the CHC of *KPC*^*TRE*^ *p48cre;LSL-Kras*^*G12D*^*;p53*^*flox/WT*^*;CHC;RIK* ES cells. The *KPC*^*shp53*^ model was generated by targeting *p48cre;LSL-Kras*^*G12D*^*;p53*^*flox/WT*^*;CHC;RIK* embryonic stem cells with a shRNA targeting mouse *Trp53* (guide strand: TTACACATGTACTTGTAGTGG, cloned into cTGM). CHC targeting of *KPC*^*cis-shRNA*^ ES cells was performed as described above using FLP-mediated recombination with cTGM vectors encoding shRNAs targeting *Renilla* luciferase to generate *KPC*^*cis-shRenilla*^ or shSmad4 (guide sequence: CAAAGATGAATTGGATTCTTT) to generate *KPC*^*cis-shSmad4*^ ES cells.

Targeting and validation was performed as described previously^31^ in M15 + LIF medium through co-electroporation (Lonza nucleofector) with shRNA encoding cTGM vectors and FLP-recombinase (CMV-flpe). Targeted cells were selected in hygromycin and resistant clones were isolated and expanded. Correct integration of targeted shRNAs was verified by the genotyping PCRs described below along with two additional tests to determine the number of integrants and conditional function. (1) The TaqMan copy-number assay was performed for shRNA-linked GFP (Invitrogen) according to the manufacturer’s instructions on a ViiA7 RT–PCR machine (Life Technologies). (2) Functionally, clones displaying single integration were treated with adenoviral Cre recombinase (University of Iowa) and grown in doxycycline-containing medium (1 μg ml^−1^) for 3 days followed by flow cytometry (Guava cytometer, Millipore; Guavasoft v.4.0) to ensure GFP expression was achieved only after Cre-mediated expression of *mKate* and *rtta*. All ES cells were confirmed to be free of mycoplasma and other microorganisms before blastocyst injection. Blastocyst injection into albino Bl6 hosts and subsequent implantation into surrogate mothers was performed as described previously^31^.

### In vivo animal studies

All of the animal experiments were performed in accordance with protocol 11-06-018 approved by the Memorial Sloan-Kettering Institutional Animal Care and Use Committee. All of the mouse strains have been previously described: the *p48-cre*^51^, *LSL-KrasG12D*^52^, *p53flox*^53^, *LSL-p53R172H*^54^, *CHC*^35^ and *CAGs-LSL-RIK*^34^ strains were maintained on mixed Bl6/129J backgrounds. Sample sizes for animal experiments were not predetermined, but all of the experiments represent a comparison of at least three different mice or the result of injection of cells from at least three different donors. Sample sizes of each experiment are noted in the figure legends. KPC^LOH^ mice were randomly distributed into groups based on sporadic tumour development. Female athymic nude mice between 6 and 8 weeks of age (Envigo) were used for orthotopic transplant experiments. BL6N mice were crossed for the generation of primary mouse embryonic fibroblasts. As described above, male ES cells were used for the generation of engineered cohorts and therefore male GEMM-ES cell mice were analysed. These male *KPC*^*LOH/shRenilla*^, *KPC*^*shp53*^, *KPC*^*cis-shRenilla*^ and *KPC*^*cis-shSmad4*^ mice were maintained on doxycycline chow (625 mg kg^−1^, replenished twice weekly, Harlan Laboratories) at 4 weeks of age until euthanasia. Athymic nude hosts were placed on doxycycline chow a week before orthotopic transplant and maintained on doxycycline chow until euthanasia. For both orthotopic or autochthonous tumour development studies, mice were immediately euthanized (end point) when the earlier of two conditions were observed: (1) the tumour size reached the limits defined in the approved animal protocol (specifically, tumours did not exceed a maximum diameter of 15 mm) or (2) if the mice presented with body condition indicative of cachexia, signs of discomfort, blood loss, abdominal distension indicative of secondary cancer stigmata like ascites, weight loss of >20% of their initial weight, severe infection, blood loss or difficulty breathing. No tumours exceeded the size limit defined in the approved animal protocol. Animals were housed on a 12 h–12 h light–dark cycle under standard temperature and humidity, at around 18–24 °C and 40–60%, respectively.

### Genotyping PCR

Genomic DNA was extracted from sorted cells and primary cultures using the QIAGEN AllPrep DNA/RNA mini kit according to the manufacturer’s instructions. Genomic DNA (10 ng) was amplified using the primers listed below with Herculase polymerase (Agilent) according to the manufacturer’s instructions:

LSL-KrasG12D-WT, *LSL* cassette, recombined: (1) 5′-GTCTTTCCCCAGCACAGTGC-3′; (2) 5′-CTCTTGCCTACGCCACCAGCTC-3′; and (3) 5′-AGCTAGCCACCATGGCTTGAGTAAGTCTGCA-3′.

Rosa26-LSL-rtta-IRES-mKate2 (RIK): (1) 5′-GGTGAGCGAGCTGATTAAGG-3′; and (2) 5′-TTTTGCTGCCGTACATGAAG-3′.

*p53*^*flox*^ WT, *flox*, recombined: (1) 5′-CACAAAAACAGGTTAAACCCAG-3′; (2) 5′-AGCACATAGGAGGCAGAGAC-3′; and (3) 5′-GAAGACAGAAAAGGGGAGGG-3′.

*CHC* WT, *Col1a1* gene, targeted *CHC*: (1) 5′-AATCATCCCAGGTGCACAGCATTGCGG-3′; (2) 5′-GGATGTGGAATGTGTGCGAG-3′; (3) 5′-ATCAAGGAAACCCTGGACTACTGCG-3′; and (4) 5′-CTTTGAGGGCTCATGAACCTCCCAGG-3′.

Original images of genotyping PCR gels are provided in Supplementary Fig. 1.

### Digital droplet PCR

DNA was extracted from flow-sorted DP and SP cells isolated from PDAC bearing *KPC*^*LOH*^ pancreata using AllPrep DNA/RNA Mini kits (Qiagen). Digital droplet PCR was performed on DNA to detect probes targeting WT *p53*, recombined *p53*^*flox*^, *CHC*, WT *Kras* and *Kras*^*G12D*^ using the T100 thermal cycler (Bio-Rad) according to the manufacturer’s instructions.

*Trp53* WT amplicon sequence and mm10 genome coordinates: TGGGAGCCGTGTCCGCGCCATGGCCATCTACAAGAAGTCACAGCACATGACGGAGGTCGTGAGACGCTGCCCCCACCTGAGCGCTGCTCCGATGGTGATGGTAAGCCCTCAACACCGCCTGT

mm10, chromosome 11: 69588447–69588569:+

*Kras*^*G12D*^ (WT/mutant) amplicon sequencing, mm10 genome coordinates: TTATTTTTATTGTAAGGCCTGCTGAAAATGACTGAGTATAAACTTGTGGTGGTTGGAGCTG[G/A]TGGCGTAGGCAAGAGCGCCTTGACGATACAGCTAATTCAGAATCACTTTGTGGATGAGTAT

mm10, chromosome 6: 145246710–145246832:−

### Primary cultures

Primary cancer cultures and cell lines were generated from the pancreas of male *KPC*^*shRenilla/LOH*^, *KPC*^*shp53*^, *KPC*^*cis-shRenilla*^ and *KPC*^*cis-shSmad4*^ mice after tumour development. Pancreas tissue was diced with scissors and digested with 1 mg ml^−1^ collagenase V (Sigma-Aldrich) diluted in Hanks buffered saline solution followed by 0.25% trypsin. Digested tissues were washed with complete DMEM (DMEM, 10% FBS (GIBCO), 1× penicillin–streptomycin) and grown in complete DMEM on collagen-coated plates (PurCol, Advanced Biomatrix, 0.1 mg ml^−1^) supplemented with 1 μg ml^−1^ doxycycline at 37 °C. Authentication of primary cultures was performed by flow cytometry of engineered fluorescent alleles and all cultures were routinely tested for mycoplasma. 

### Cell sorting and flow cytometry

Mouse pancreatic tissue for sorting or flow cytometry (as well as from orthotopic transplant) was processed as described previously^55^. In brief, pancreatic tissue was gently minced with scissors washed with Hanks Buffered Saline solution, and then dissociated by incubation with collagenase V (1 mg ml^−1^; Thermo Fisher Scientific; in Hanks buffered saline solution), then trypsin (0.05%) and finally dispase (2 U ml^−1^, Invitrogen). DNase I (100 μg ml^−1^, Sigma-Aldrich) was added during all enzyme incubations. Cells were washed with PBS between the collagenase and trypsin steps, and with FACS buffer (2% FBS, 10 mM EGTA, in PBS) between the trypsin and dispase steps. Suspensions were then filtered through a 40 μm mesh and resuspended in FACS buffer with 300 nM DAPI for bulk or single-cell sorting into 96-well plates on Aria3 sorters (BD, maintained by the MSKCC Flow Cytometry Facility; FACS Diva v.8.0). Flow cytometry from dissociated pancreata after growth of tumours following orthotopic transplant and from primary cultures at the indicated timepoints was performed on Fortessa instruments (BD, maintained by the MSKCC Flow Cytometry Facility; FACS Diva v.8.0). Ploidy profiling was performed using NST-DAPI buffer as described previously^56^ on an Attune NxT flow cytometer (Invitrogen; Attune NxT v.3.1). Human PDAC flow cytometry and ploidy profiling for single-cell sequencing is described in more detail below. For calculating the median ploidy of tumour polyploid distributions, the ratio of the median DAPI-area measurements for diploid and polyploids gates was calculated and multiplied by 2 (for example, assuming the diploid distribution has a median ploidy of 2). Supplementary Fig. 2 illustrates the gating strategy based on mKate and GFP and DAPI fluorescence.

### Histology, immunofluorescence and immunohistochemistry

Tissues were fixed overnight at 4 °C in 10% formalin before paraffin embedding and sectioning performed by IDEXX/RADIL. H&E staining was performed using conventional protocols by IDEXX/RADIL. For immunofluorescence staining, 5 μm sections were deparaffinized and rehydrated with a histoclear/alcohol series and antigen retrieval was performed by boiling in 1× citrate antigen retrieval buffer (Vector). Slides were blocked in PBS with 5% BSA and primary antibody staining was performed overnight in blocking buffer at 4 °C. For IHC staining endogenous peroxidases were blocked after antigen retrieval by treating slides with 3% hydrogen peroxide for 15 min at room temperature. The following primary antibodies were used: chicken anti-GFP (1:500, Abcam, 13970), rabbit anti-mKate2/Turbo RFP (1:1,000, Evrogen, AB233), mouse anti-Ki-67 (1:500, BD, 550609), rabbit anti-p53 (1:100, NCL-L-p53-CM5p, Leica Biosystems). Primary antibodies were detected using the following fluorescently conjugated secondary antibodies at a 1:500 dilution: goat-anti-chicken AF488 (Invitrogen, A32931), goat anti-rabbit 555 (Invitrogen, A32732), goat anti-mouse AF633 (Invitrogen, A-21052). All secondary antibodies were diluted in blocking buffer and incubated for 1 h at room temperature. Stained slides were washed and nuclei were counterstained with PBS containing DAPI and mounted under cover slips with ProLong Gold (LifeTechnologies). Labelling of primary antibodies for DAB staining was performed using the IHC ImmPRESS HRP Goat Anti-Rabbit IgG Polymer Peroxidase Detection Kit (VectorLabs, MP-7451) for 1 h at room temperature and developed with DAB reagent (VectorLabs, SK-4100). IHC sections were counterstained with haematoxylin and mounted with VectorMount (VectorLabs, H-5000-60) after dehydration. Images were acquired using the Zeiss AxioImager microscope using Zeiss ZEN 3.3 software. Scanning of slides for matched histology (H&E) and immunofluorescence was performed using the Aperio Versa system (Leica) with the Aperio VERSA Application (v.1.0.4) software.

### Low-density-sorted growth assay

A total of 500 DP or SP cells sorted from tumour or non-tumour bearing mice were sorted as described into 500 μl of complete DMEM. Cells were transferred to PurCol-coated (as described above) 12-well plates and the cell number was counted after 2 weeks of growth using a Guava flow cytometer (Millipore software, GuavaSoft v.4.0).

### Orthotopic transplant assays

DP and SP cells were sorted from tumour- and non-tumour-bearing mice as described. For tumour-bearing mice, 10,000 or 25,000 cells were sorted into FACS buffer (PBS, 2% FBS); for non-tumour bearing mice, 100–1,000 SP cells were sorted into FACS buffer (PBS, 2% FBS). For comparison with SP cells sorted from non-tumour-bearing mice, 100 SP cells from tumour-bearing mice were sorted. Cells were washed with PBS, resuspended in 25 μl of a 1:1 mix of serum-free DMEM and growth-factor-reduced Matrigel (Corning), and injected into the exposed pancreas of athymic nude mice using a Hamilton syringe fitted with a 26-gauge needle. Recipient mice were enrolled on doxycycline chow (625 mg kg^−1^, Harlan Laboratories) 2 days before surgery and maintained on doxycycline chow until euthanasia due to tumours reaching the IACUC approved humane end points for tumour burden (see above). If tumours did not form, the mice were monitored until they were euthanized due to age-related decline in health.

### DNA-FISH analysis

DNA fluorescence in situ hybridization (DNA-FISH) analysis was performed on paraffin sections using a three-colour probe designed to detect copy-number changes of chromosome 2, 9 and 10. The bacterial artificial chromosome clones used in the probe mix were based on sequencing data (minimal region of gain/loss) and are as follows: 9qC (RP23-248H6, RP23-340D4, RP23-60M16; labelled with Spectrum Red) and 2qC (RP23-332C13, RP23-186P20, RP23-435A5; labelled with Spectrum Green). All RP11 clones were purchased from the Roswell Park Cancer Institute Genomics Shared Resource. Probe labelling, hybridization, post-hybridization washing and fluorescence detection were performed according to procedures established at the Molecular Cytogenetics Core Facility. Slides were scanned using a Zeiss Axioplan 2i epifluorescence microscope (Carl Zeiss Microscopy) equipped with Isis imaging software (MetaSystems). The entire section was first scanned through 63× to assess signal pattern. Corresponding H&E and/or immunostained slides were used to identify regions of premalignant or cancer morphology (foci of adenocarcinoma). Regions selected for analysis were imaged through the depth of the tissue (compressed stack of 12 *z*-sections at 0.5 μm intervals) and, for each case, within each representative region at least 50 discrete nuclei were scored.

### Matched immunofluorescence and histology quantification

For quantification of immunofluorescence staining of Ki-67 in mKate/GFP DP and mKate SP cells in PDAC-bearing mice, 5 random ×10 images were collected in grossly premalignant and PDAC tissue areas. For quantification of immunofluorescence staining of Ki-67 in mKate/GFP DP and mKate SP cells in mice before PDAC development, ×20 fields containing all SP cells without PDAC morphology observed in one pancreatic cross-section were collected from nine mice. Three of these mice also displayed focal areas of incipient PDAC where up to 5 random ×20 fields were collected. Tissue sections from the pancreas of tumour- and non-tumour-bearing mice were simultaneously stained for GFP, mKate2, Ki-67 and DAPI. Quantification of the Ki-67 fraction in DP and SP cells was conducted using a semi-automated strategy. First, images were background-subtracted and processed using a Gaussian filter using MATLAB. Second, nuclei (DAPI^+^) and epithelial (mKate2^+^ and/or GFP^+^) cells were identified using Otsu-based segmentation in MATLAB. Third, epithelial cells were classified as either SP or DP on the basis of mKate2 and GFP status. Fourth, cells were nominated as Ki-67^+^ by applying a fixed threshold, determined by aggregating data from all of the fields of view. Finally, assignment of Ki-67 status was manually corrected by inspecting each image individually. In the case of fields containing rare pre-tumour SP lesions in which DP cells vastly outnumbered SP cells, DP cells were randomly subsampled to match the number of SP cells that were found in the same field of view. Fields of view in which there were no DP or SP cells were excluded from the analysis. For both of these analyses Wilcoxon’s rank-sum test was used to compare the groups.

For determination of the frequency of mKate/GFP DP and mKate SP cells in histopathologically defined premalignant and PDAC cells, lesions were identified from up to 20 random fields on H&E-stained slides from 6 PDAC-bearing pancreata. Lesions of interest were classified by pathologists blinded to immunofluorescence data. Lesions defined as ADM were identified in fields of 4 out of 6 mice analysed and lesions defined as PanIN were identified in fields of 5 out of 6 mice.

For histological classification of mKate SP cells in mice before PDAC development (Fig. 1g), 43 SP-cell-containing lesions were identified in cross-sections of seven pancreata without frank PDAC using immunofluorescence for mKate and GFP as described. These lesions were then identified on sequential, H&E-stained sections and classified by pathologists who were blinded to the immunofluorescence data.

For DNA-FISH quantification based on morphology and *p53* genotype, first FISH for chromosomes 2, 9 and 10 was performed on 5 pre-tumour *KPC*^*LOH*^ pancreata as described above. FISH images were collected of fields containing either SP cells with PDAC or premalignant morphology based on H&E and fluorescence staining of sequential sections. Next, cover slips were removed from FISH-stained slides and, after washing with PBS for 1 h at 4 °C to remove mounting medium, slides were processed for immunofluorescence staining for mKate and GFP as described. FISH foci were then scored in 50 discrete cells in verified SP cells with PDAC or premalignant morphology.

### LMD analysis

Regions for LMD were identified from sequential sections of formalin-fixed paraffin-embedded tissues stained for mKate, GFP and DAPI as described above. Sections for LMD were placed on membrane slides (PPS-Membrane FrameSlides, 11600294, Leica) and subjected to rehydration and light haematoxylin staining. Epithelial regions corresponding to mKate/GFP DP or mKate SP immunofluorescence signal were cut using hand-drawn regions of interest and collected in PCR tubes with an LMD7000 laser microdissection microscope (Leica) using the Leica micro-dissection software (v.7.5.1). DNA was isolated using the Qiagen QIAamp DNA FFPE Advanced Kit (56604) according to the manufacturer’s recommended protocol.

### TCGA PDAC SNP array data and human/mouse synteny analysis

Processed, segmented single-nucleotide polymorphism (SNP) array data were download from the TCGA Genomics Data Commons Data Portal (https://portal.gdc.cancer.gov/). To filter out spurious single-probe segments, array probe coordinates were converted to genomic bin coordinates with a 5 bin threshold used to accept/reject a segment. Frequency plot analysis was performed on the transformed dataset (*n* = 186 patient samples) by aggregating bin values and accepting a threshold values of ±0.1 for gain/deletion designation, respectively, in each bin. Approximately 66% of samples contained *TP53* point mutations that were classified as missense compared to truncating, splice site or structural variant somatic variants. Of the missense classified mutations, approximately 30% affected residues designated as hotspots (such as Arg175, Arg248 and Arg273—the three most frequent sites). Human–mouse synteny analysis was performed as described previously^57^. Human reference human build hg19 and mouse reference genome build mm9 were used for the analysis while selecting a resolution of 400 kb of contiguous sequence.

### *KPC*^*LOH*^ bulk, LMD and single-cell sequencing

DP and SP cells were disaggregated from PDAC and non-tumour bearing pancreas and processed for flow cytometry as described above. For bulk SP/DP sorting from PDAC samples, the instrument was set in high-purity mode with cells pelleted after cytometry and processed for DNA/RNA extraction using the AllPrep DNA/RNA Mini kit (Qiagen) according to the manufacturer’s instructions. Subsequently, 500 ng of genomic DNA was processed for library generation as previously described^58^. For single-cell deposition and whole-genome amplification (WGA) from tumour as well as pre-tumour samples, single cells were sorted using the instrument set on single-cell mode with realignment/calibration of the automated cell deposition unit and a wash step performed before processing each unique sample. Single cells (DP and SP) were deposited into wells of a 96-well plate prepared with 9 μl of lysis buffer as described previously^56^. The number of single cells sequenced per sample category (for example, tumour or pre-tumour) is summarized in Supplementary Table 1. For single-cell WGA, we used a modified version of the DOP-PCR WGA, a WGA approach that has been empirically determined to yield highly accurate single-cell copy-number data^59–61^. The modified approach introduces inline barcode sequences during the second step of the WGA amplification reaction. After WGA, single-cell WGA DNA products were purified using the QIAquick 96 PCR purification kit according to the manufacturer’s recommendations. Purified DNA was subsequently quantified with equal amounts of WGA DNA (100 ng) per cell pooled and processed for Illumina library sequencing preparation using the NEBNext kit (NEB). For sequencing of LMD material, eluted DNA was subject to the WGA protocol and downstream sequence library preparation as described above. Sequencing libraries were sequenced on the HiSeq2500 instrument using single-end 101 bp reads while targeting an average coverage of 1 million reads per single-cell, a coverage that was previously determined to be sufficient for accurate genome-wide copy-number determination at a bin resolution of 600 kb^58^. A minimum of 250,000 reads per cell was required for inclusion in downstream analyses.

### *KPC*^*LOH*^ bulk, LMD and single-cell sequencing data analysis

Bulk, LMD, and single-cell sequencing data were mapped to mouse reference genome build mm9 while skipping the first 50 bp containing inline barcoding sequences as well as the DOP-PCR quasi-degenerate sequence. Uniquely mapped reads were indexed, sorted and PCR duplicates were subsequently removed. Uniquely mapped sequencing reads were counted in genomic bins/intervals that were computed using a previously developed algorithm (Varbin)^62^. The genome was partitioned into 5,000 bins of approximately 600 kb in length. Read counts were subsequently corrected for GC content using the LOWESS smoothing algorithm^63^, normalized and segmented using circular binary segmentation^64^. Ploidy was inferred using a least-squares fitting algorithm while factoring integer bin value assignment distribution^58^. For downstream single-cell analysis, absolute copy number calls below or above a reference normal (copy number = 2 diploid single cells and copy number = 4 for polyploid single cells) were defined as deletions and gains, respectively, using the algorithm CORE^65^. Datasets were similarly processed using an orthogonal copy number calling algorithm (HMMcopy^47^). Results were highly concordant between the two analysis pipelines for both human and mouse datasets and are illustrated in Supplementary Fig. 3. For bulk frequency plot analysis, normalized segments for all of the samples were centred around a mean value of 1 with thresholds of ±0.1 used for defining gained/deleted bins. For illustration of single-cell copy-number profiles across sequence samples, heat maps of PDAC (SP and DP) and pre-tumour SP single cells were constructed using absolute copy-number values imputed from the single-cell data and clustered using the Ward method with Manhattan disease.

### Aggregate single-cell genotyping for *p53* LOH haplotype inference

A hybrid genome of mm10 with the sequence elements for eGFP and mKate was made and then indexed for BWA. Raw fastq data were first mapped to the hybrid genome using bwa and then counts of reads mapping to the two trans-elements (eGFP and mKate) along with genes *Clp1* and *Trp53* were collected using bedtools coverage. Reads were filtered to remove supplementary alignments, those marked with alternative hits (XA:Z: bwa flag) and those with a MAPQ < 30. The resulting coverage files were then processed with R scripts to normalize the counts to RPKM values and then plot the distribution of normalized coverage by gene and sample type.

### Phylogenetic analysis of *KPC*^*LO*H^ single-cell sequencing data

Phylogenetic and clonal relationships between DP and SP cells from PDAC and pre-tumour mice were investigated using two orthogonal methods: a change-point/breakpoint-based analytical method^66,67^ as implemented using the SCC lust software package (available at GitHub/KrasnitzLab) and a minimum-event distance (MED) method that models whole-genome duplication events in reconstructing phylogenies and ancestral genomes (MEDICC2^68^). In brief, SCClust identifies change points throughout a set of integer-valued copy-number profiles of single-cell genomes. For all pairs of cells, dissimilarity is quantified using Fisher’s exact test for change-point coincidence and used to cluster the cell genomes hierarchically. Nodes of the tree with a statistically significant high degree of similarity among the leaves are identified as sampled from clonal cell populations. For this purpose, statistical significance of dissimilarity within pairs of cells is quantified by the FDR, in comparison to the null distribution of dissimilarities following a random interchange of change points among cells. SCClust was used with the default parameters except for the ‘keepboundaries’ parameter, which was set to True. Figure 3a was prepared using a previously described visualization tool (Single-Cell Genome Viewer)^66^ available from GitHub/KrasnitzLab. In MEDCICC2, copy-number segments are presented as positive integer vectors with the algorithm solving for MED between pairs of copy-number profiles (for example, profile = aggregate segments of a single cell). The algorithm also models whole-genome duplication events using a set of constraints, including accounting for chromosome boundaries as described in ref. ^68^. MEDICC2 constructs phylogenetic trees on the basis of imputed MED values over single cells while minimizing the total number of events that explains a transformation of one profile into another. A total of 200 iterations of resampling bootstrap were used in assigning confidence in branching relationships between diploid and polyploid single cells. MEDICC2 provided support tree panels with bootstrap confidence statistics on branch/node relationships are available in Supplementary Figs. 4 and 5 for pre-tumours 1 and 3, respectively. Owing to the lack of heterozygous SNPs in the genome of inbred laboratory mice of C57BL/6NJ strain background (from which the *KPC*^*LOH*^ model was constructed) compared to humans, MEDICC2 was run using total copy number without phase information using flag --total-copy-numbers. Compared to an average human individual with around 1.5 million heterozygous SNPs, the average number of heterozygous SNPs of an inbred C57BL/6NJ mouse is 16,000. This statistic was retrieved from the Sanger Mouse Genomes Project (ftp://ftp-mouse.sanger.ac.uk/REL-1303-SNPs_Indels-GRCm38/mgp_v3_stats.txt).

### Human PDAC whole-genome sequencing data analysis

WGS data of microdissected pancreatic tumours were downloaded from EGA (accession EGAD00001006152)^23^. WGS data were subsequently downsampled to a depth of around 10 million sequencing reads with the data processed for copy-number analysis as previously described^56^. In brief, sequencing reads were mapped to human reference genome hg19 with uniquely mapped reads sorted and indexed. Uniquely mapped reads were subsequently counted in genomic bins while partitioning the genome in 20,000 bins using a previously described algorithm (Varbin)^62^. Read bin counts were normalized genome-wide with subsequent processing using circular binary segmentation^64^. For absolute copy-number quantification, segmented data were processed using a least-squares fitting algorithm^58^ with the parameters of the algorithm constrained by ploidy values derived from a previously published algorithm (CELLULOID)^23^. As performed for mouse single-cell data, sequencing reads were similarly processed using HMMcopy with the results largely concordant as shown in Supplementary Fig. 6. *TP53* mutation type statistics were similar to those described for the TCGA cohort (discussed above). Absolute copy-number data were subsequently partitioned into three categories for downstream analysis: diploid/*TP53* monoallelic/WT, diploid/*TP53*-biallelic and polyploid/*TP53*-biallelic. For *TP53-*mutation allelic status associative analysis with ploidy, Fisher exact tests were used for significance. For *TP53* allelic and ploidy categories, we derived significant recurrent CNAs using the algorithm CORE^65^, which computes scores and genomic coordinates for significantly recurrent CNAs with scores proportional to their occurrence in a given dataset (that is, patient cohort). Copy-number calls of less or greater than a reference normal/pseudonormal (2 for diploid; 4 for polyploid) were classified as deletion or gain events, respectively. Statistical tests for the enrichment of event types were based on *t*-sample *t*-tests for significance. For analysis of the level of heterogeneity/homogeneity of deletions and gains, FACETS^69^ (https://github.com/mskcc/facets/; version Oct 8, 2021; commit 3058bba) was used to process whole-genome sequencing data. To generate the SNP count files, a snp-pileup command was run using a cleaned version of dbSnp v.137 (filter to contain only SNV and with duplicates removed). The resulting tumour/normal pileups were then processed using the standard FACETS method as described in the manual. Specifically: mat = readSnpMatrix(countFile), xx = preProcSample(mat,snp.nbhd = 1000), oo = procSample(xx,cval = 500), fit = emcncf(oo). The inferred heterogeneity/homogeneity of chromosomal segments was computed by taking the estimated cell fraction (cf) statistic of each segment relative to the computed purity of the sequenced sample (for example, cf/purity) and analysing the distribution of the resulting statistic using a Kolmogorov–Smirnov test. A two-sample Kolmogorov–Smirnov test was used to determine the significance of the difference between the distributions.

### MSK-IMPACT target capture data analysis and clinical correlations

A total of 1,077 PDAC cases sequenced using MSKCC institutional targeted sequencing panel (MSK-IMPACT) were analysed. Zygosity determination, genome-wide total and allele-specific DNA copy number, purity and ploidy were calculated using FACETS (v.0.5.13)^69^. The expected number of copies for each mutation, used in designating TP53 allelic state, was generated on the basis of the observed variant allele fraction and local ploidy^70^. Cancer cell fractions were calculated using a binomial distribution and maximum likelihood estimation normalized to produce posterior probabilities^71^. Inferred heterogeneity/homogeneity of chromosomal segments was performed as described above and similarly tested for statistical significance using a two-sided Kolmogorov–Smirnov test. Clinical annotations of disease spread and patient outcome were performed manually and subsequently used in genomic correlative analysis. For significant of correlation between ploidy status and disease spread, Fisher exact tests were implemented. Univariate Cox regression analysis was used to ascertain prognostic predictive value of disease spread.

### Human single-cell sequencing and data analysis

Frozen tissue from nine resected PDACs (four diploid and five polyploid) that were biallelically mutant for *TP53* were processed for single-nucleus isolation as previously described^56^. Research involving resected samples was approved by the MSKCC institutional review board (MSKCC IRB). In brief, two 50 μm tissue sections were cut and mechanically dissociated using a pair of scalpels in the presence of 1 ml of nuclei isolation buffer (NST-DAPI). DAPI-stained nuclei were subsequently sorted on the basis of DNA content with single-nuclei collected from the diploid and polyploid gate (when present) and deposited into 96-well plates preloaded with nucleus lysis buffer as described above. Single-nucleus WGA amplification was performed as described above for mouse SP and DP single cells with downstream sequence library performed using the NEBNext Library preparation kit (NEB). Libraries were sequenced on the HiSeq2500 instrument for single-end 101 bp sequencing while targeting a coverage of around 1–2 million reads per single cell. Single-cell demultiplexed sequencing data were processed for absolute copy-number determination as previously described^58^. The absolute copy number was converted to the nearest integer, and homogeneity score was inferred as the frequency of the major integer copy-number state for each bin across the genome.

### Materials availability

Plasmids, mouse ES cells and primary cancer cell lines generated and used in this work are available from the corresponding author.

### Reporting summary

Further information on research design is available in the Nature Research Reporting Summary linked to this article.

## Online content

Any methods, additional references, Nature Research reporting summaries, source data, extended data, supplementary information, acknowledgements, peer review information; details of author contributions and competing interests; and statements of data and code availability are available at 10.1038/s41586-022-05082-5.

## Supplementary information

Supplementary Information Supplementary Figs. 1–6.

Reporting Summary

Supplementary Table 1

## Data Availability

All human and mouse sequencing data generated in this study are publicly available at the Sequence Read Archive (SRA; https://www.ncbi.nlm.nih.gov/sra) under accession number PRJNA718334. Human bulk PDAC sequencing data are available at the European Genome–Phenome Archive (EGA; https://www.ebi.ac.uk/ega/) under accession code EGAD00001006152. EGA data are accessible for research purposes by registration for an EGA account and contacting the Data Access Committee. Source data are provided with this paper.

## Source data

Source Data Fig. 1
Source Data Fig. 4
Source Data Fig. 5
Source Data Extended Data Fig. 1
Source Data Extended Data Fig. 2
Source Data Extended Data Fig. 7
Source Data Extended Data Fig. 8

## Code availability

All core code used in this study have been previously published with downloadable and runnable source codes available at GitHub (https://github.com/robertaboukhalil/ginkgo; https://github.com/KrasnitzLab/SCGV; https://github.com/KrasnitzLab/SCclust). The full breakpoint-based phylogenetic analyses including data and code is available online (https://github.com/KrasnitzLab/p53-LOH-figures; https://hub.docker.com/repository/docker/krasnitzlab/p53-loh-figures). For Docker, free registration and installation is required (https://www.docker.com). Analysis and plotting scripts relating to heat maps and heterogeneity/homogeneity metrics are available at GitHub (https://github.com/naikai/p53-LOH-figures).
