# Supplementary material for: Ordered and deterministic cancer genome evolution after p53 loss
Source: Nature. 2022 Aug 17;608(7924):795–802. doi: 10.1038/s41586-022-05082-5 (PMC9402436; doi:10.1038/s41586-022-05082-5)
Supplement: Supplementary file 1 — Supplementary Figs. 1–6. [file 41586_2022_5082_MOESM1_ESM.pdf]

nature portfolio

https://doi.org/10.1038/s41586-022-05082-5

Supplementary information

Ordered and deterministic cancer genome evolution after p53 loss

In the format provided by the authors and unedited

Ordered and deterministic cancer genome evolution after p53 loss.

Timour Baslan, John P. Morris IV, Zhen Zhao, Jose Reyes, Yu-Jui Ho, Kaloyan M. Tsanov, Jonathan Bermeo, Sha Tian, Sean Zhang, Gokce Askan, Aslihan Yavas, Nicolas Lecomte, Amanda Erakky, Anna M. Varghese, Amy Zhang, Jude Kendall, Elena Ghiban, Lubomir Chorbadjiev, Jie Wu, Nevenka Dimitrova, Kalyani Chadalavada, Gouri J. Nanjangud, Chaitanya Bandlamudi, Yixiao Gong, Mark T.A. Donoghue, Nicholas D. Socci, Alex Krasnitz, Faiyaz Notta, Steve D. Leach, Christine A. Iacobuzio-Donahue, and Scott W. Lowe

Supplementary Information

Page 1: Table of contents
Page 2: Supplementary Figure 1 | Uncropped gel electrophoresis images of genotyping PCRs.
Page 3: Supplementary Figure 2 | Flow cytometry gating strategy.
Page 4: Supplementary Figure 3 | Concordance of copy number inference from mouse and human single cell sequencing data between orthogonal computational tools.
Page 5: Supplementary Figure 4 | Algorithm provided support tree panel with bootstrap confidence statistics on branch/node relationships for KPC^{LOH} Pre-Tumor 1.
Page 6: Supplementary Figure 5 | Algorithm provided support tree panel with bootstrap confidence statistics on branch/node relationships for KPC^{LOH} Pre-Tumor 3.
Page 7: Supplementary Figure 6 | Concordance of copy number inference from bulk pancreatic cancer whole genome sequencing data using orthogonal computational tools.

Supplementary Data Figure 1 | Uncropped gel electropheresis images of genotyping PCRs.

Extended Data Figure 1F

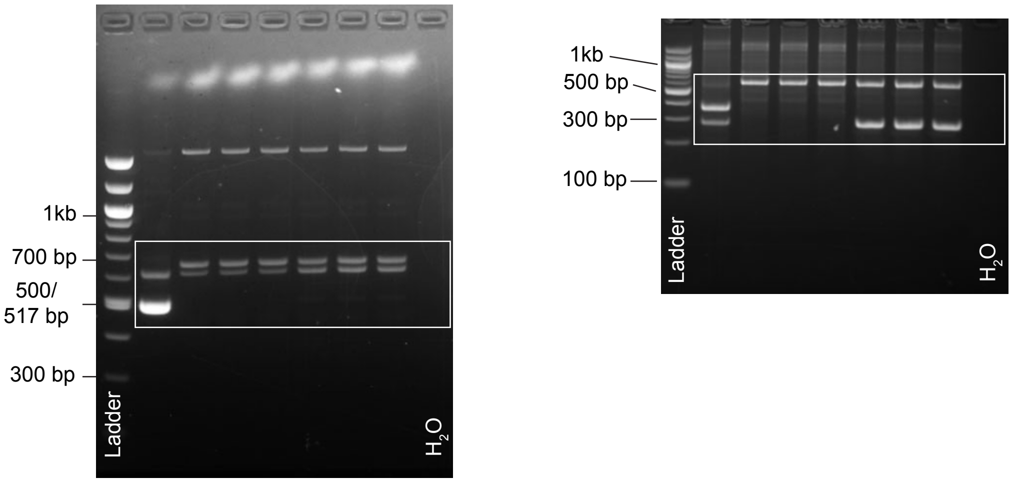

Extended Data Figure 1G

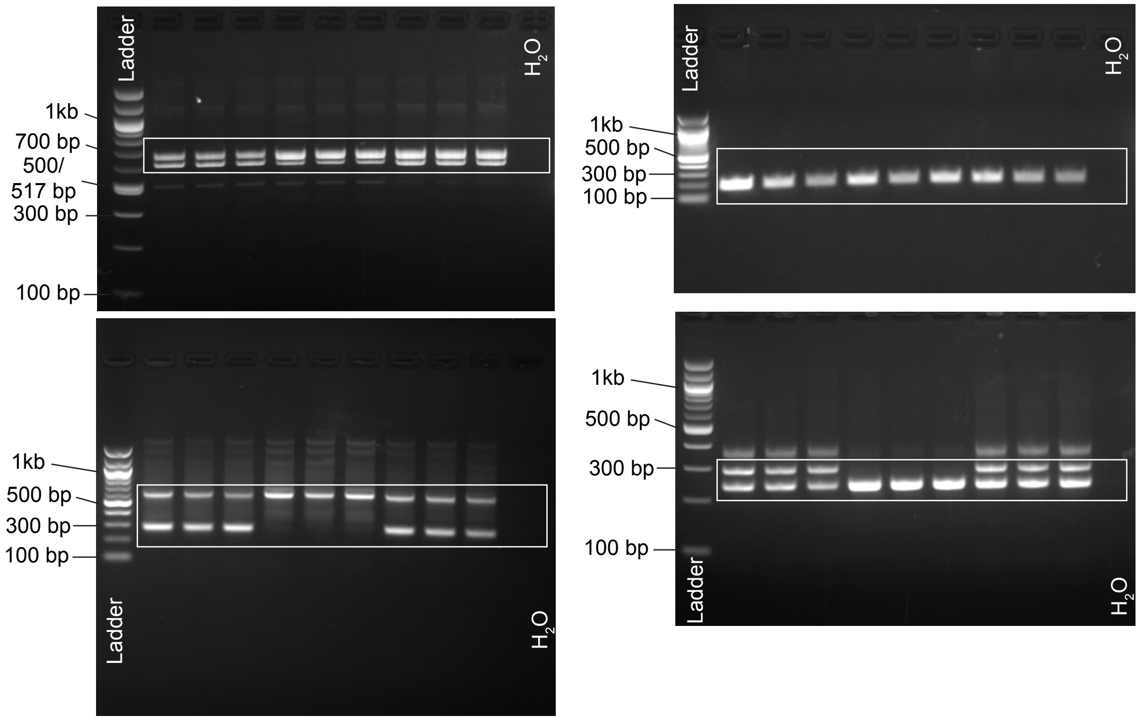

Extended Data Figure 2C

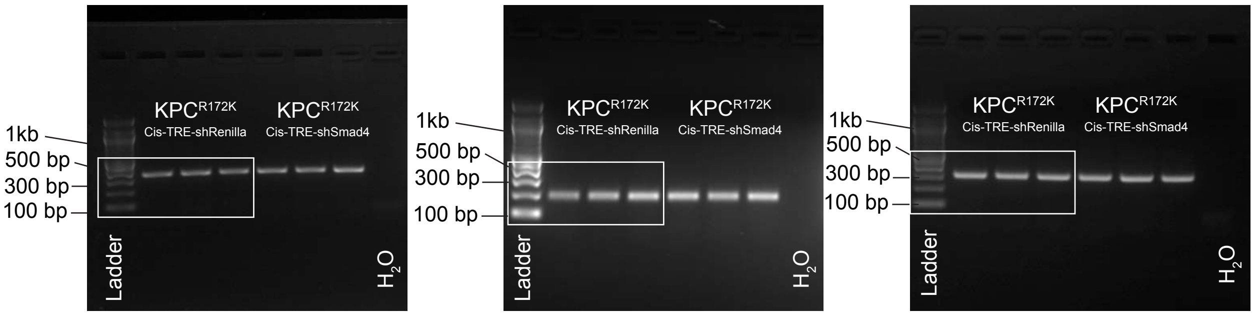

Supplementary Figure 1 | Uncropped gel elecropheresis images of genotyping PCRs. Excised/cropped images are denoted in white boxes. Ladder sizes indicated in base pairs (bp).

Supplementary Data Figure 2 | Flow cytometry gating strategies.

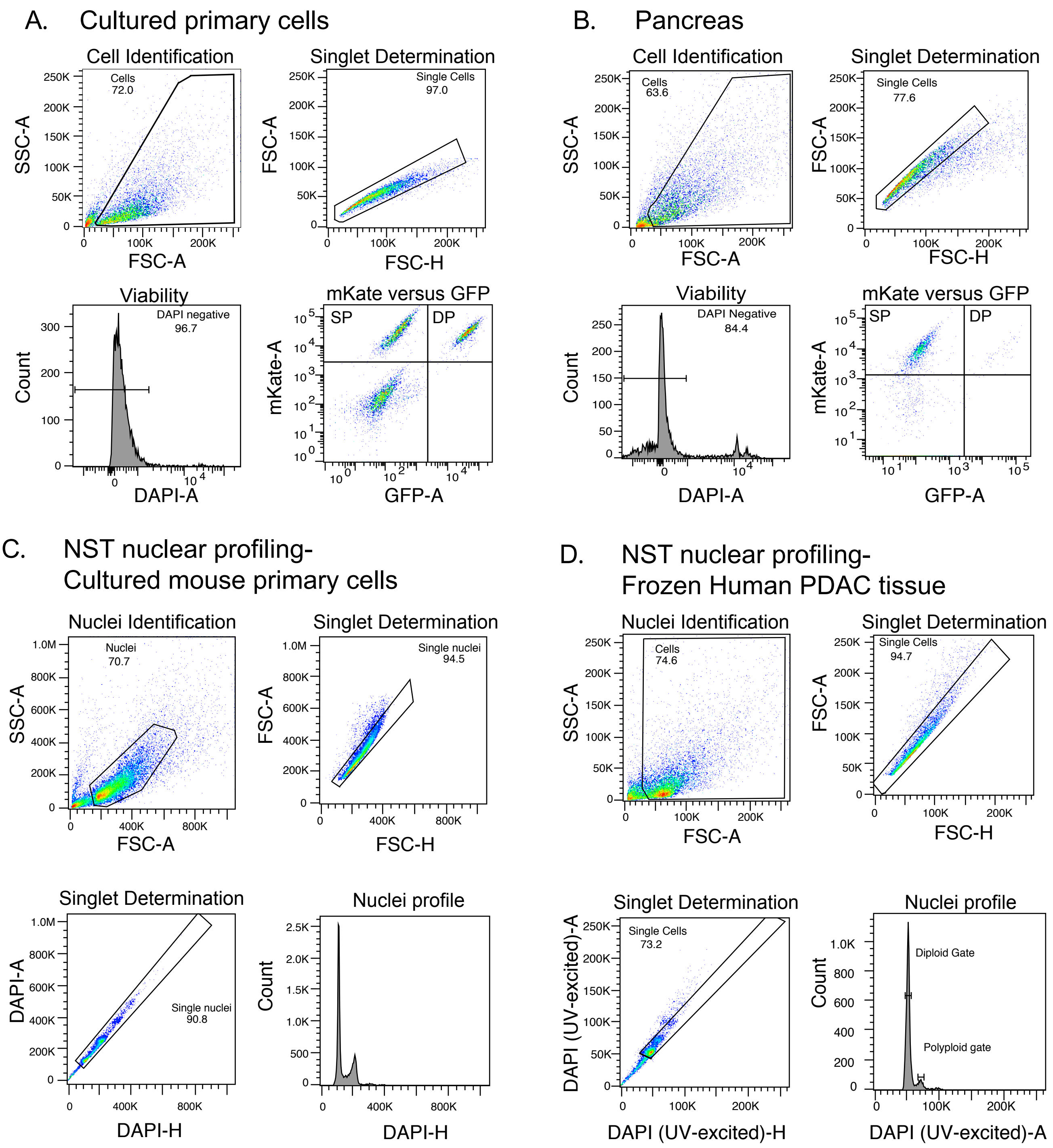

Supplementary Figure 2 | Flow cytometry gating strategies. a, Cultured primary cells. **b**, Dissociated KPC^{LOH} tumor bearing mice. **c,** NST-DAPI nuclei profiling of cultured mouse primary cells sorted from KPC^{LOH} mice. **d**, NST-DAPI nuclei profiling of frozen human PDAC tissue. Gates identifying diploid and poylploid fractions are indicated.

Supplementary Data Figure 3 | Concordance of copy number inference from mouse and human single cell sequencing data using orthogonal computational tools.

A.

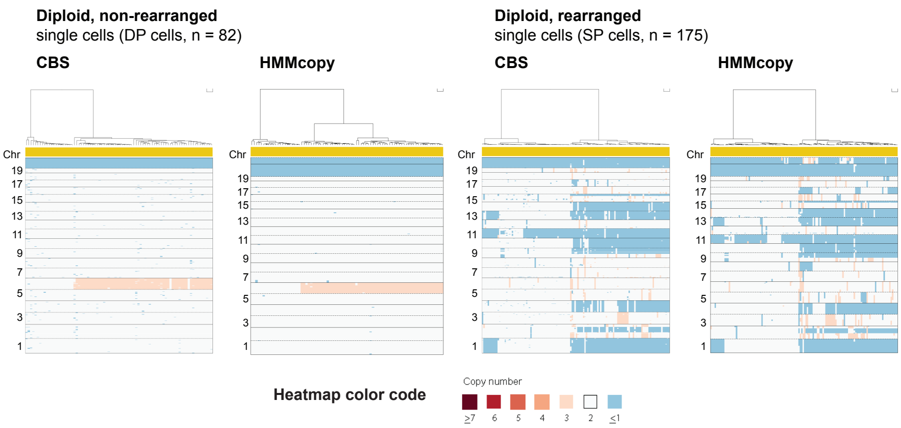

B.

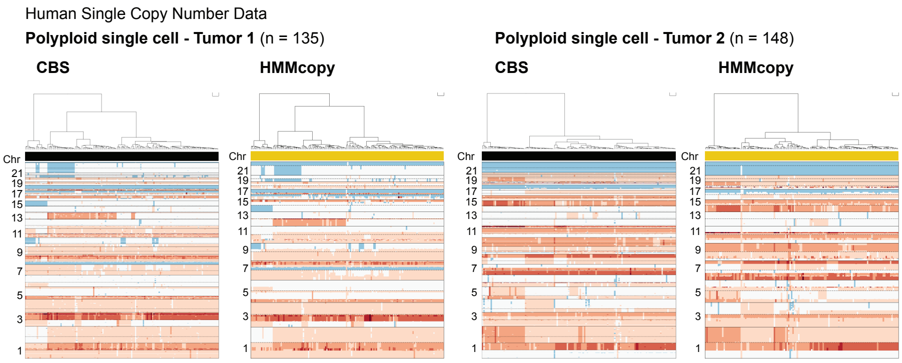

Supplementary Data Figure 3 | Concordance of copy number inference from mouse and human single cell sequencing data between orthogonal computational tools. a, Comparison of copy number inference from mouse non-rearranged diploid (left panel) and rearrange diploid (middle panel). Number of sequenced and analyzed single cells is provided. Heatmap color code is provided. **b**, Comparison of copy number inference from two human polyploid PDACs sequenced at single cell resolution using CBS and HMMcopy algorithms. Number of sequenced and analyzed single cells is provided. Heatmap color code is provided.

support tree

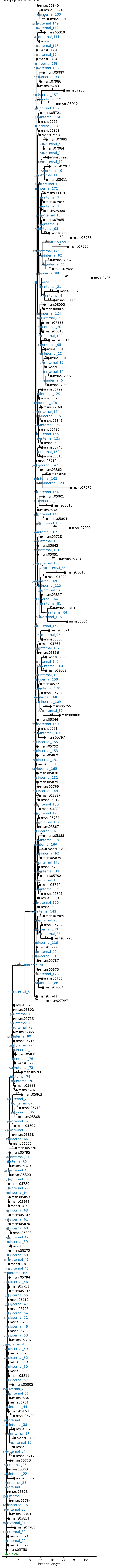

support tree

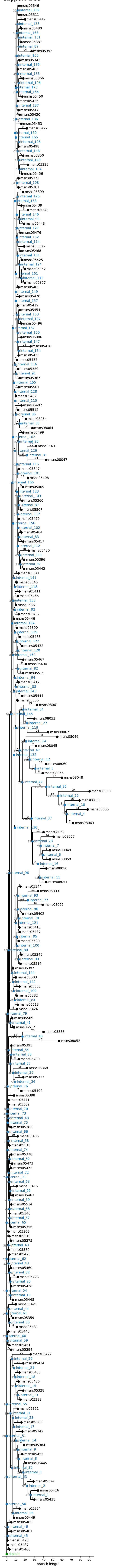

Supplementary Data Figure 4 | Concordance of copy number inference from bulk pancreatic cancer whole genome sequencing data using orthogonal computational tools.

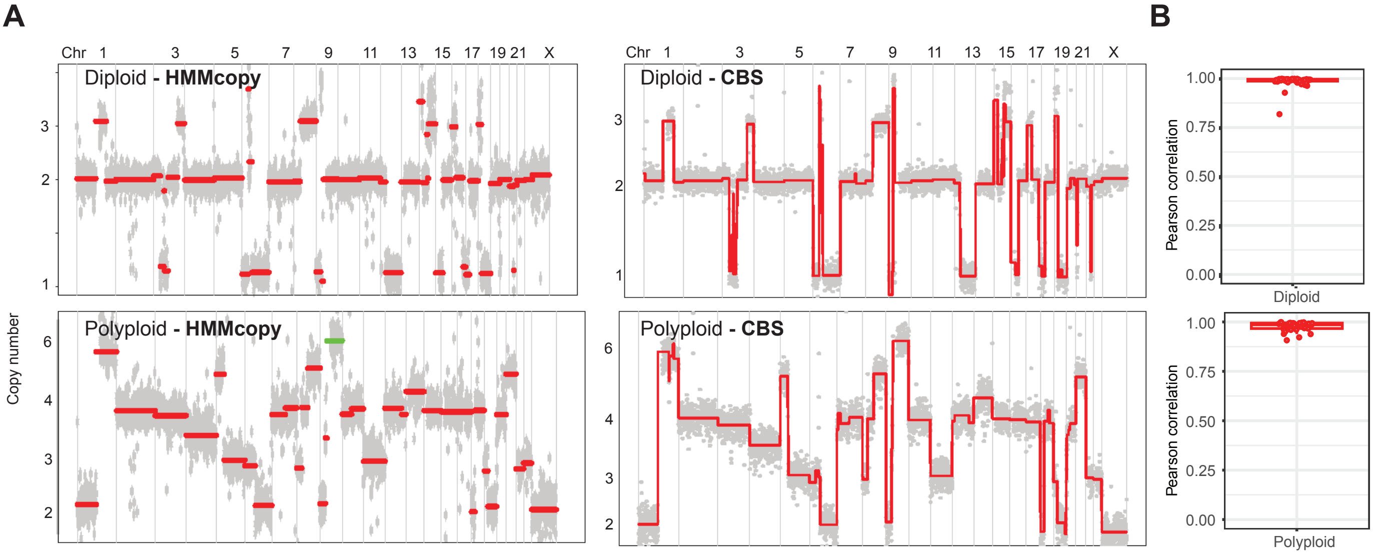

Supplementary Data Figure 4. Concordance of copy number inference from bulk pancreatic cancer whole genome sequencing data using orthogonal computational tools. a, Representative example of a comparison of whole genome copy number inference from a diploid (top panels) and a polyploid (bottom panels) PDAC analyzed using two orthogonal algorithms; HMMCopy (left panels) and CBS (right panels). **b**, Pearson correlation values of inferred copy number events across the COMPASS dataset for diploid (top panels) and polyploid (bottom panel) PDACs.
