# Supplementary material for: Ordered and deterministic cancer genome evolution after p53 loss
Source: Nature. 2022 Aug 17;608(7924):795–802. doi: 10.1038/s41586-022-05082-5 (PMC9402436; doi:10.1038/s41586-022-05082-5)
Supplement: Supplementary file 2 — Reporting Summary [file 41586_2022_5082_MOESM2_ESM.pdf]

nature portfolio

Corresponding author(s): Scott Lowe

Last updated by author(s): Jun 18, 2022

Reporting Summary

Nature Portfolio wishes to improve the reproducibility of the work that we publish. This form provides structure for consistency and transparency in reporting. For further information on Nature Portfolio policies, see our Editorial Policies and the Editorial Policy Checklist.

Statistics

For all statistical analyses, confirm that the following items are present in the figure legend, table legend, main text, or Methods section.

n/a	Confirmed	
☐	☒	The exact sample size (*n*) for each experimental group/condition, given as a discrete number and unit of measurement
☐	☒	A statement on whether measurements were taken from distinct samples or whether the same sample was measured repeatedly
☐	☒	The statistical test(s) used AND whether they are one- or two-sided<br>*Only common tests should be described solely by name; describe more complex techniques in the Methods section.*
☒	☐	A description of all covariates tested
☐	☒	A description of any assumptions or corrections, such as tests of normality and adjustment for multiple comparisons
☐	☒	A full description of the statistical parameters including central tendency (e.g. means) or other basic estimates (e.g. regression coefficient) AND variation (e.g. standard deviation) or associated estimates of uncertainty (e.g. confidence intervals)
☐	☒	For null hypothesis testing, the test statistic (e.g. *F*, *t*, *r*) with confidence intervals, effect sizes, degrees of freedom and *P* value noted<br>*Give P values as exact values whenever suitable.*
☒	☐	For Bayesian analysis, information on the choice of priors and Markov chain Monte Carlo settings
☒	☐	For hierarchical and complex designs, identification of the appropriate level for tests and full reporting of outcomes
☐	☒	Estimates of effect sizes (e.g. Cohen's *d*, Pearson's *r*), indicating how they were calculated

Our web collection on statistics for biologists contains articles on many of the points above.

Software and code

Policy information about availability of computer code

Data collection	Histology (H&E), immunofluoresence, and immunohistochemistry images were collected with Zeiss ZEN 3.3 software. Whole section histology and fluorescent images were collected with Aperio VERSA Application Software v1.0.4. Flow cytometry and sorting was performed with FACS DIVA v8.0 and additional flow cytometry for nuclear content performed with Attune NxT software version 3.1. Laser microdissection was performed with Leica micro-dissection software V7.5.1.
Data analysis	All core code utilized in this study have been previously published and is referenced in the Methods section. Links to repositories including new custom analysis scripts are provided in the Methods section.

For manuscripts utilizing custom algorithms or software that are central to the research but not yet described in published literature, software must be made available to editors and reviewers. We strongly encourage code deposition in a community repository (e.g. GitHub). See the Nature Portfolio guidelines for submitting code & software for further information.

Data

Policy information about availability of data

All manuscripts must include a data availability statement. This statement should provide the following information, where applicable:

- Accession codes, unique identifiers, or web links for publicly available datasets
- A description of any restrictions on data availability
- For clinical datasets or third party data, please ensure that the statement adheres to our policy

All human and mouse sequencing data generated in this study are publicly available via Short Read Archive (SRA - https://www.ncbi.nlm.nih.gov/sra) accession PRJNA718334.
Human bulk PDAC sequencing data is available via European Genome-Phenome Archive (EGA - https://www.ebi.ac.uk/ega/) accession code EGAD00001006152.

EGA Data is accessible for research purposes via registration for an EGA account and contacting the Data Access Committee.

A link to the full breakpoint based phylogenetic analyses including data and code can be found at:
https://github.com/KrasnitzLab/p53-LOH-figures
https://hub.docker.com/repository/docker/krasnitzlab/p53-loh-figures
For Docker, free registration and installation is required (https://www.docker.com)

Field-specific reporting

Please select the one below that is the best fit for your research. If you are not sure, read the appropriate sections before making your selection.

☒ Life sciences ☐ Behavioural & social sciences ☐ Ecological, evolutionary & environmental sciences

For a reference copy of the document with all sections, see nature.com/documents/nr-reporting-summary-flat.pdf

Life sciences study design

All studies must disclose on these points even when the disclosure is negative.

Sample size	For biological characterization (e.g. tissue analysis, functional experiments performed on isolated cells) we set out to achieve sample sizes comparing at least 3 biological replicates as recognized as a conventional minimum for identifying meaningful differences. As indicated in the figure legends these experiments were performed with samples sizes between 4 and 8 and in the case of survival studies of genetically engineered mice developing tumors at least 20. For sequencing studies, sample sizes reflected those utilized in biological characterization of the mouse model.
Data exclusions	No tissue analysis or biological functional data were excluded. For sequencing, only single cells that did not meet criteria as defined in the Methods section were excluded.
Replication	Whole cross section matched immunohistochemistry/immunofluorescence analyses were repeated at least 3 times. Immunohistochemical staining was robust across experiments performed by different investigators. Functional analyses (e.g. immunohistochemical/ immunofluorescence based quantification, tumor initiation studies via transplantation or growth of isolated cells) were performed with multiple biological replicates as indicated in figure legends and methods section. For genotyping experiments where n=3 representative samples are shown, the experiments were repeated at least twice with similar results and were cross validated in independent biological replicates by orthogonal experiments (e.g. digital droplet PCR, analysis of single cell sequencing data). Matched immunofluorescence and p53 immunohistochemistry was repeated 3 times on tissues from different PDAC bearing KPCLOH mice with similar results. For sequencing, each single cell served as an independent biological replicate from each unique biological sample.
Randomization	Samples were randomly distributed into groups.
Blinding	Pathologists were blinded to lineage tracing information when evaluating the patho-histology of the mouse model as described in the methods. Investigators were not blinded to other analysis of the lineage tracing model facilitated by its fluorescence because of the inherent link between fluorescent color and genomic status.

Reporting for specific materials, systems and methods

We require information from authors about some types of materials, experimental systems and methods used in many studies. Here, indicate whether each material, system or method listed is relevant to your study. If you are not sure if a list item applies to your research, read the appropriate section before selecting a response.

Materials & experimental systems

n/a	Involved in the study	
☐	☒	Antibodies
☐	☒	Eukaryotic cell lines
☒	☐	Palaeontology and archaeology
☐	☒	Animals and other organisms
☒	☐	Human research participants
☐	☒	Clinical data
☒	☐	Dual use research of concern

Methods

n/a	Involved in the study	
☒	☐	ChIP-seq
☐	☒	Flow cytometry
☒	☐	MRI-based neuroimaging

Antibodies

Antibodies used	Antibody, Provider, Catalogue number, Clone info-Immunogen (if provided), Lot (if provided)<br>GFP, Abcam 13970 No clone info provided-Raised against full length GFP protein, GR236651-23<br>Mkate, Evrogen, AB233, No clone info provided-Raised against full-length recombinant denatured and non-denatured TagRFP, 23301040466<br>Ki67, BD, 550609, B56

p53, Leica Biosystems, NCL-L-p53-CM5p, CM5
Goat anti-Chicken IgY (H+L) Cross-Adsorbed Secondary Antibody, Alexa Fluor™ Plus 488, Invitrogen, A32931, raised against purified chicken IgY, heavy and light chains
Goat anti-Rabbit IgG (H+L) Highly Cross-Adsorbed Secondary Antibody, Alexa Fluor™ Plus 555, Invitrogen, A32732, raised against rabbit gamma Immunoglobins heavy and light chains
Goat anti-Mouse IgG (H+L) Highly Cross-Adsorbed Secondary Antibody, Alexa Fluor™ 633, Invitrogen, A-21052, raised against mouse gamma immunoglobins heavy and light chains

Validation

Antibody, Validation and references
GFP, Manufacturer's website states validation by western blot and immunocytochemistry/immunofluorescence. Used previously for immunofluoresence in PMID 24395249
Mkate, manufacturer's website states validation by western blot, immunoblotting, immunocytochemistry, and ELISA. Used previously for immunofluoresence in PMID 24395249
Ki67, manufacturer's website states validation by immunohistochemistry. Previously used for immunofluorescence in PMID 31534224
p53, manufacturer's website states validation by western blot. Previously used for western blot in the context of a shRNA in PMID 31534224

Eukaryotic cell lines

Policy information about cell lines

Cell line source(s)

KPCshRenilla-SP cells grown from dissociation of PDAC bearing KPCLOH pancreas and KPCshp53 cells grown from dissociation of PDAC bearing KPCshp53 pancreas. KPCshRenilla-SP cells sorted from mice without frank PDAC, grown out from plating at low density. KPC-cis-shRenilla and KPC-cis-shSmad4 cells grown from dissociation of PDAC bearing mice. Primary mouse embryonic fibroblasts grown from ~e13.5 Bl6N embryos.

Authentication

Cell lines were not externally authenticated.

Mycoplasma contamination

Cells routinely tested negative for mycoplasma contamination.

Commonly misidentified lines
(See ICLAC register)

No cell lines used in this study were found in the database of commonly misidentified cell lines that is maintained by ICLAC and NCBI Biosample.

Animals and other organisms

Policy information about studies involving animals; ARRIVE guidelines recommended for reporting animal research

Laboratory animals

p48-Cre; LSL-KrasG12D; p53flox; Rosa26-CAGs-LSL-rtta-IRES-mKate2 ; Col1a1-TRE-shRenilla (KPCLOH); p48-Cre; LSL-KrasG12D; p53flox; Rosa26-CAGs-LSL-rtta-IRES-mKate2 ; Col1a1-TRE-shp53 (KPCshp53); p48-Cre; LSL-KrasG12D; p53LSL-R172H; Rosa26-CAGs-LSL-rtta-IRES-mKate2 ; Col1a1-TRE-shRenilla (KPCcis-shRenilla); p48-Cre; LSL-KrasG12D; p53LSL-R172H; Rosa26-CAGs-LSL-rtta-IRES-mKate2 ; Col1a1-TRE-shSmad4 (KPCcis-shSmad4) were all male, generated from male ES cells derived from blastocysts resulting from breeding of constituent alleles maintained on mixed, BL6/129 backgrounds with shRNA constructs targeted to the CHC. All nude mice used for transplant experiments were adult 6-8 weeks old females. Pregnant Bl6N female mice were sacrificed for collection of embryos for primary MEFs. Mouse housing details are provided in the methods section.

Wild animals

No wild animals were used in this study.

Field-collected samples

No field collected samples were used in this study.

Ethics oversight

MSKCC IACUC. All experiments were performed under protocol #11-06-018

Note that full information on the approval of the study protocol must also be provided in the manuscript.

Clinical data

Policy information about clinical studies
All manuscripts should comply with the ICMJE guidelines for publication of clinical research and a completed CONSORT checklist must be included with all submissions.

Clinical trial registration

Provide the trial registration number from ClinicalTrials.gov or an equivalent agency.

Study protocol

Note where the full trial protocol can be accessed OR if not available, explain why.

Data collection

Describe the settings and locales of data collection, noting the time periods of recruitment and data collection.

Outcomes

Describe how you pre-defined primary and secondary outcome measures and how you assessed these measures.

Flow Cytometry

Plots

Confirm that:

☒ The axis labels state the marker and fluorochrome used (e.g. CD4-FITC).

☒ The axis scales are clearly visible. Include numbers along axes only for bottom left plot of group (a 'group' is an analysis of identical markers).

☒ All plots are contour plots with outliers or pseudocolor plots.

☒ A numerical value for number of cells or percentage (with statistics) is provided.

Methodology

Sample preparation	For flow cytometry of live cells grown in cell culture dishes, cells were washed with PBS, trypsinized to generate a single cell suspension, filtered through 40uM mesh capped tubes to eliminate cell clumps, and resuspended in full media with DAPI to discriminate live and dead cells. For flow cytometry of cells from tumors, single cell suspensions were generated by enzyme digestion as described in the methods, filtered through 40uM mesh, and resuspended for analysis in FACs buffer with 300nM DAPI to discriminate live and dead cells. For NST nuclear profiling cells were cells were washed with PBS, trypsinized to generate a single cell suspension, filtered through 40uM mesh capped tubes to eliminate cell clumps an resuspended in NST-DAPI buffer as described in the Methods.
Instrument	BD LSRFortessa and Attune NxT for cytometry, BD Aria III for sorting
Software	FACS DIVA (BD) was used for data acquisition, FlowJo (FLowJo LLC) was used for analysis. No custom code was used for analysis.
Cell population abundance	Where possible, (e.g. sorted cell growth experiments) post sort fluorescence was verified by fluorescence microscopy. When cell number permitted, post sort analysis was performed on a small number of sorted cells to ensure purity. Purity of post sort populations was >90%.
Gating strategy	For all flow cytometry experiments cells were first identified by FSC/SSC such that all cells were visible on the plot. Single cells were gated using Area versus Height analysis. DAPI positive cells were excluded and GFP mKate fluorescence analyzed. For NST experiments singlets were discriminated by comparing FSC-A versus FSC-H then DAPI-A versus DAPI H.

☒ Tick this box to confirm that a figure exemplifying the gating strategy is provided in the Supplementary Information.
